# *Mycobacterium tuberculosis* complex lineage 5 exhibits high levels of within-lineage genomic diversity and differing gene content compared to the type strain H37Rv

**DOI:** 10.1099/mgen.0.000437

**Published:** 2021-07-09

**Authors:** C. N'Dira Sanoussi, Mireia Coscolla, Boatema Ofori-Anyinam, Isaac Darko Otchere, Martin Antonio, Stefan Niemann, Julian Parkhill, Simon Harris, Dorothy Yeboah-Manu, Sebastien Gagneux, Leen Rigouts, Dissou Affolabi, Bouke C. de Jong, Conor J. Meehan

**Affiliations:** ^1^​ Laboratoire de Référence des Mycobactéries, Cotonou, Benin; ^2^​ Mycobacteriology Unit, Institute of Tropical Medicine, Antwerp, Belgium; ^3^​ Department of Biomedical Sciences, University of Antwerp, Antwerp, Belgium; ^4^​ I2SysBio, University of Valencia-FISABIO Joint Unit, Valencia, Spain; ^5^​ Food and Drugs Authority, Accra, Ghana; ^6^​ Rutgers New Jersey Medical School, Rutgers University, New Jersey, USA; ^7^​ Noguchi Memorial Institute for Medical Research, University of Ghana, Legon, Accra, Ghana; ^8^​ Medical Research Council Unit in The Gambia at the London School of Hygiene and Tropical Medicine, Fajara, The Gambia; ^9^​ German Center for Infection Research, partner site Borstel-Hamburg-Lübeck-Riems, Borstel, Germany; ^10^​ Research Center Borstel, Molecular and Experimental Mycobacteriology, Borstel, Germany; ^11^​ Wellcome Sanger Institute, Hinxton, UK; ^12^​ Department of Veterinary Medicine, University of Cambridge, Cambridge, UK; ^13^​ Swiss Tropical and Public Health Institute, Basel, Switzerland; ^14^​ University of Basel, Basel, Switzerland; ^15^​ School of Chemistry and Biosciences, University of Bradford, Bradford, UK

**Keywords:** genomic diversity, gene presence/absence, H37Rv, lineage 5, L5.3.2, *M. africanum*, reference genome, within-lineage variability

## Abstract

Pathogens of the *Mycobacterium tuberculosis* complex (MTBC) are considered to be monomorphic, with little gene content variation between strains. Nevertheless, several genotypic and phenotypic factors separate strains of the different MTBC lineages (L), especially L5 and L6 (traditionally termed *Mycobacterium africanum*) strains, from each other. However, this genome variability and gene content, especially of L5 strains, has not been fully explored and may be important for pathobiology and current approaches for genomic analysis of MTBC strains, including transmission studies. By comparing the genomes of 355 L5 clinical strains (including 3 complete genomes and 352 Illumina whole-genome sequenced isolates) to each other and to H37Rv, we identified multiple genes that were differentially present or absent between H37Rv and L5 strains. Additionally, considerable gene content variability was found across L5 strains, including a split in the L5.3 sub-lineage into L5.3.1 and L5.3.2. These gene content differences had a small knock-on effect on transmission cluster estimation, with clustering rates influenced by the selected reference genome, and with potential overestimation of recent transmission when using H37Rv as the reference genome. We conclude that full capture of the gene diversity, especially high-resolution outbreak analysis, requires a variation of the single H37Rv-centric reference genome mapping approach currently used in most whole-genome sequencing data analysis pipelines. Moreover, the high within-lineage gene content variability suggests that the pan-genome of *M. tuberculosis* is at least several kilobases larger than previously thought, implying that a concatenated or reference-free genome assembly (*de novo*) approach may be needed for particular questions.

## Data Summary

Sequence data for the Illumina dataset are available at the European Nucleotide Archive (ENA; https://www.ebi.ac.uk/ega/) under the study accession numbers PRJEB9003, PRJEB38656, PRJEB4884, PRJEB38317, PRJEB31139, PRJEB6273 and PRJEB31144. Individual run accession numbers are indicated in Table S8 (available in the online version of this article). PacBio raw reads for the L5 Benin genome are available on the ENA accession SAME3170744. The assembled L5 Benin genome is available at the National Center for Biotechnology Information (NCBI) with accession PRJNA641267. The two other complete genomes of L5 strains from Gambia (PcbL5Gam, WBB1453_11-00429-1 [1]) and Nigeria (PcbL5Nig, WBB1454_IB091-1 [1]) can be found at http://pathogenseq.lshtm.ac.uk/#tuberculosis (Tuberculosis section/Karonga Methylation study). To ensure that the naming conventions for the genes in the genomes of the three L5 strains can be followed, we have uploaded these annotated files to figshare [2].

The custom Python scripts used in this analysis can be found at https://github.com/conmeehan/pathophy.

**Impact Statement**

The *Mycobacterium tuberculosis* complex (MTBC) consists of nine human-associated lineages. Although many of these have been described for decades, little is known about the gene content variation both between and within strains of these lineages. This is most pronounced for strains of lineage 5 (L5), once part of the *Mycobacterium africanum* species. We compared the genomes of over 350 clinical L5 strains, the largest dataset gathered to date, to each other and the H37Rv reference strain to look for gene content variation and the potential impact this would have on clinical use of genome sequence data. We found that multiple genes are differentially present or absent between H37Rv and L5 strains, and that there is high within-L5 gene content variability, resulting in the split of the sub-lineage L5.3 into L5.3.1 and L5.3.2. We quantified the potential impact of this gene content difference on transmission clustering estimation. We found that the current H37Rv-centric approach widely used in MTBC epidemiology would overestimate the clustering rate of L5 strains since it misses single-nucleotide polymorphisms present in L5-only genes. Thus, for high-resolution outbreak analysis, MTBC epidemiological studies may need to move away from the H37Rv-centric approach, especially when looking at transmission in countries where L5 is prominent.

## Introduction

Tuberculosis (TB) is caused by pathogenic bacteria of the *Mycobacterium tuberculosis* complex (MTBC) that consists of strains of nine human-adapted lineages and several animal-adapted lineages [3–6]. This group is highly clonal with no detected horizontal gene transfer [7, 8]. Strains of particular lineages are primarily defined by large sequence polymorphisms (LSPs, the presence or deletion of genomic regions) such as the TbD1 region (MTBC-specific deletion 1) [9], other regions of difference (RDs) [10, 7, 11–13] and signature single-nucleotide polymorphisms (SNPs) [14]. RDs are particular long genomic regions deleted in some groups of MTBC strains but present in others; thus many MTBC lineages can be classified in groups using that LSP [10, 12, 13, 15]. Broadly, the lineages of the MTBC occur within three major clades: (1) L1–L4 and L7 form one group, (2) L5, L6, L9 and the animal lineages form another and (3) L8 sits within its own clade [4–6], based on the presence/absence of specific RDs, especially TbD1 [12, 16, 17]. L5 and L6, also called *M. tuberculosis var*. *africanum* (or historically *M. africanum* West-african 1 and 2, respectively) [18, 19] are primarily restricted to West Africa, where they cause up to 40 % of human TB [20, 21]. The reasons for the geographical restriction of L5 and L6 remain unclear, although adaptation to particular human subpopulations has been suggested [10, 22–24].

Several phenotypic and genotypic features separate L5 and L6 strains from strains of the other human-adapted lineages. Some TB diagnostics have a lower performance for L5 and L6 strains, compared to strains of other MTBC lineages [25, 26] and these lineages are less likely to grow in culture [27], with dysgonic appearance on solid medium [18, 26, 28, 29], despite the inclusion of pyruvate-supplemented medium [21, 27]. Mutations in genes essential for growth in culture were identified for L6 strains [30], yet for L5 strains the reasons for the difficulty in growth remain unclear. L6 strains and those of the closely related animal strains/lineages such as *Mycobacterium bovis* are reported to be less virulent in humans than those from other human-adapted MTBC lineages in population-based studies [20, 31, 32] and in genome studies of mutations in virulence regulation genes/systems [33, 34]. Infection with an L6 strain progresses slowly to TB disease, and is associated with impaired immunity in some settings but not all (e.g. HIV infection [20]). In contrast, although some authors have studied the genomics of L5 strains [35–37], protein secretion and *in vivo* immunogenicity [35], much remains to be learned on the genomics, virulence and disease progression of L5 strains.

Whole-genome sequencing (WGS) based on next-generation sequencing (NGS) of MTBC strains often involves data analysis by mapping of short sequence reads of the strains to a complete reference genome [38], usually H37Rv [39, 40] or a reconstructed ancestor with the same gene content as H37Rv [22, 41, 42]. The resulting SNPs are then used for drug resistance determination, subtyping and transmission analyses [38]. However, since H37Rv is a L4 strain, it might not be representative for the genome of strains of other MTBC lineages. Thus, if H37Rv is used as basis for genome analysis, several genes or larger genomic regions may be missed, resulting in an underestimation of genome diversity among the strains of other lineages that may, however, provide additional information, e.g. for transmission analysis.

The members of the MTBC evolved from an environmental organism to an obligate pathogen through genome reduction and acquisition of new genes [43]. In addition, it is known that some differences in gene content exist between strains of different lineages [10, 38, 44–48]. Furthermore, it was reported that genes had a higher genetic diversity among L6 strains compared to L5 ones [36], but the difference in gene content between strains of these two lineages was not investigated, but had been alluded to in an earlier abstract publication [37]. Further, little is known about the gene differences between genomes of L4 and L5 strains and the potential limitations this may impose for in-depth analysis of genome studies of L5 strains (e.g. sub-lineage detection and transmission tracking). Similarly, little is known about within-lineage diversity in terms of gene content.

To address these questions, we assessed the gene content diversity of L5 strains in the context of WGS and reference selection. To this end, we analysed 358 genomes of L5 strains, including 3 complete genomes, and compared them to H37Rv and strains of closely related lineages [L6 and *M. tuberculosis var*. bovis (hereafter called *M. bovis*) [19]. Our main focus was to determine particularities in the genomes of L5 strains compared to the MTBC strain type (H37Rv, a L4) and strains of MTBC lineages phylogenetically closely related to L5 (L6 and *M. bovis*); deduce hypotheses for L5 phenotype/biology; and define the level of within-lineage gene content differences among strains of this lineage of the MTBC.

## Methods

### Genomes

#### Complete genomes (PacBio-sequenced, complete long reads)

Three complete genomes from L5 clinical isolates, all sequenced with the PacBio SMRT technology, were analysed. One genome was from a Benin isolate [PcbL5Ben; sequenced in this study, National Center for Biotechnology Information (NCBI) accession PRJNA641267], one from an isolate from The Gambia (PcbL5Gam, WBB1453_11-00429-1) [1] and one from Nigeria (PcbL5Nig, WBB1454_IB091-1) [1]. These genomes also represent disparate parts of L5’s diversity, representing a broad range of L5 sub-lineages, as recently defined [6]. The previously published reference/complete genomes H37Rv (L4) (NC_000962.3) [39, 40, 49], L6 (GM041182, GenBank accession: FR878060.1, GCF_0001593225.1_ASM159322v1) [50] and *M. bovis* (AF2122/97, accession: LT708304.1) [51] were also included.

#### Whole genomic DNA extraction

Genomic DNA extraction was performed on growth from fresh Löwenstein–Jensen slants using the semi-automated Maxwell 16 Cell DNA purification kit in the Maxwell 16 machine, or from the late exponential phase of growth in 7H9 medium, using the CTAB method [6, 52].

#### Assembly and annotation of the complete (PacBio-sequenced) genomes

The Benin PacBio-sequenced genome was assembled using HGAP [53] and Quiver [53] and checked for sufficient quality and coverage (10 000 bp sliding window coverage was always above 65× and average coverage across entire genome was 107×). The other two PacBio-sequenced genomes were already assembled as described previously [1]. The genome sequences of all three complete (PacBio-sequenced) genomes were annotated using Prokka-1.12 [54] based on the reference genome H37Rv annotation.

#### Gene presence–absence analysis

Gene content differences were assessed with an all-vs-all blastn approach, using blast+ version 2.8.1 [55, 56]. For a specific genome–genome comparison, the following procedure was used: the gene sequences of genome 1 (ffn file; coding regions only) were compared to those of genome 2 using an *e*-value cut-off of 1e^−05^ and a minimum similarity of 70 % to look for any homology for each gene. Those genes found in genome 1 and not in genome 2 were then compared to the complete (PacBio-sequenced) genome (fna file; coding and non-coding regions) of genome 2 to look for pseudogenes (herein qualified as ‘suspected pseudogenes’) using blastn with the same cut-offs. These suspected pseudogenes were then confirmed using tblastn of the genome 1 protein sequences (faa file) compared to the complete (PacBio-sequenced) genome (fna file) of genome 2. This procedure was used to compare the complete (PacBio-sequenced) genomes of all three L5 strains to each other as well as each to H37Rv. Those genes present or absent in H37Rv (or pseudogenes in either) were compared in a similar manner to the L6 and *M. bovis* reference strains to determine whether these genes/pseudogenes are L5-specific (i.e. present in L5, but not in H37Rv, L6 and *M. bovis*).

The ffn files of H37Rv, the complete (PacBio-sequenced) genomes of the three L5 strains and the complete genome of the L6 reference strain were also aligned using progressiveMauve (v20150226) [57] to identify rearrangements and examine synteny.

#### L5 Illumina-sequenced (short reads) genomes

In total, whole genomes from 355 L5 strains from various countries sequenced on the Illumina platform were included in the study. These genomes were derived from a larger study on the genetic diversity of L5 and L6 [6]. After reducing isolate redundancy (i.e. 1 representative retained for those that were extremely closely related), genomes from 205 L5 strains formed a non-redundant dataset. These 205 strains (genomes) originated from West, South, East and Central Africa (Table S8), but primarily (*n*=155) from two regions within West Africa: the western part of West Africa (including The Gambia, Sierra Leone, Ivory Coast, Liberia, Guinea and Mali) and the eastern part of West Africa (including Ghana, Benin and Nigeria) [6]. In general, L5 and L6 are geographically restricted to West and Central Africa, making this genome selection from L5 strains representative of most of the geographical regions affected.

#### Mapping of L5 Illumina reads to H37Rv and L5 complete (PacBio-sequenced) genomes

Raw reads (fastq files) of the 205 non-redundant Illumina-sequenced genomes were mapped respectively to H37Rv and each of the PacBio-sequenced genomes of the 3 L5 strains using the MTBseq pipeline [58]. The depth mapping coverage of the samples in the clinical Illumina-sequenced genomes against the reference genomes (percentage read mapping, unambiguous coverage mean) was compared between the PacBio-sequenced genomes of the three L5 strains and between the PacBio-sequenced genome of each L5 strain and H37Rv. Mapping statistics parameters such as percentage unambiguous total base, uncovered bases, SNPs, deletions, insertions, substitutions and percentage genes mapped to reference were also compared using the PacBio-sequenced genome of each L5 strain or H37Rv as a reference.

#### Checking unique versus missing genes in Illumina-sequenced genomes of L5 strains and SNPs in those confirmed as L5-specific

The genes found to be present or absent in L5 based on genome comparisons of the PacBio-sequenced genomes of the three L5 strains with H37Rv were checked for their expected presence or absence in the Illumina-sequenced genomes of L5 strains. Using the position tables produced by the MTBseq pipeline, a gene was considered absent if 95 % of its position in the genome had fewer than eight reads covering them. From these data, a gene presence/absence matrix was generated for Illumina-sequenced genomes of L5 strains mapped to H37Rv and each of the three complete (PacBio-sequenced) genomes of L5 strains. Genes found to be L5-specific (present in complete PacBio- and Illumina-sequenced genomes) were also checked for SNPs in these genes using each of the complete (PacBio-sequenced) genomes of the three L5 strains as a reference. The script used for undertaking this analysis can be found at https://github.com/conmeehan/pathophy.

#### Calculating the effect of reference genome selection on pairwise SNP distances

Transmission analysis of MTBC strains often involves clustering strains together based on specific SNP cut-offs [38, 59, 60]. We assessed whether the selection of the reference genome (H37Rv, PcbL5Ben, PcbL5Gam or PcbL5Nig) changed the clustering rate of L5 strains. For this we used the non-redundant set of L5 genomes from a previous study on L5 diversity [6], which included the 205 detailed above and a further 145 strains that were closely related to at least 1 of these 205 strains. Additionally, the TB-Profiler online SRA search tool (https://tbdr.lshtm.ac.uk/sra) was used to identify a further 5 isolates that were not in this dataset, resulting in 355 strains being included in this clustering study.

SNP alignments of the Illumina-sequenced genomes were created by first mapping to each of the four reference genomes (H37Rv and three PcbL5). The Amend function of MTBseq does this automatically for H37Rv, including masking of repetitive regions, accounting for 10 % of the genome [40], and exclusion of columns with 95 % ambiguous calls. To undertake this for the reference genomes from the three L5 strains, repetitive regions were first determined from the annotations of the genes. All genes whose description contained one of the following words was excluded: integrase, PE family, PE-PGRS, phage, transposase. SNP alignments were then created using MTBseq as done for H37Rv. Pairwise distance matrices, one for each alignment based on each reference genome, were created using a Python script that can be found at https://github.com/conmeehan/pathophy. Loose transmission clusters were created from these matrices at a cut-off of 1, 5 and 12 SNPs, as described previously [59]. Clustering rates and the presence of a strain in a transmission cluster was then compared across the four reference genome mapping approaches.

#### Determination of putative function of genes differentially present or absent in the complete (PacBio-sequenced) genomes of L5 strains

To find the (putative) function of genes present or absent only in L5 strains, and genes present in L5 strains but pseudogenes in H37Rv/L6/*M. bovis* or vice versa, the fasta sequences of the genes were searched against the NCBI’s NR database using blastx2.8.1+ [61] as well as the Tuberculist database (http://tuberculist.epfl.ch), Mycobrowser (https://mycobrowser.epfl.ch/) [62] and the literature. Furthermore, the gene function group/class was found using the COG database [63] and Mycobrowser.

#### Determination of the sub-lineage of the L5 strains

The sub-lineage of the strains was determined by looking for sub-lineage-defining SNPs in the genomes of L5 strains as previously described [6, 35]. The sub-lineage-defining SNPs were searched for in the SNP data generated using the MTBseq pipeline with H37Rv as the reference genome.

## Results

### Comparative analysis of the complete (PacBio-sequenced) genomes of L5 and other lineages strains reveals differences in gene content

The number of genes, including paralogues, in the complete L5 (PacBio-sequenced) genomes of the three L5 strains was: 4189 in the PcbL5Ben genome, 4162 in PcbL5Gam and 4134 in PcbL5Nig versus 4126 in H37Rv, 4126 in the reference L6 genome (GM041182) and 4059 in the reference *M. bovis* genome (AF2122/97). Hence, the maximum gene count difference was 55 genes among the 3 L5 strains from this study (4189–4134=55, including copies of genes with multiple copies), 63 genes among the human-adapted lineages (H37Rv, L5, L6; 4189–4126=63) and 130 genes among the human- and animal-adapted lineages (H37Rv, L5, L6 and *M. bovis*; 4189–4059=130).

The size of the complete genomes of L5 strains was respectively 4 438 262 bp for PcbL5Ben, 4 424 447 for PcbL5Gam and 4 417 534 for PcbL5Nig (data in Figshare [2]). The size was 4 411 532 bp for H37Rv and 4 493 502 for the *M. bovis* reference genome (data from annotation with Prokka).

The visualization of the structure of the genomes showed that all three complete genomes of all L5 strains had a region that was absent in H37Rv. Furthermore, the PacBio-sequenced genome of the Nigerian L5 strain (PcbL5Nig) was missing an additional region (herein called L5Nig-Del) that was present in the genomes of the Benin and the Gambian L5 strains and also present in H37Rv.

The genome of the Benin strain contained a syntenic block of three genes (1148, 1104, 2840 bp) that were present in neither the genome of the Nigerian strain nor the one of the Gambian strain (Tables 1 and 2). These increased the pangenome of L5 by at least 5092 bp. The Benin and Gambian strains each contained 33 genes that were not present in the Nigerian strain (Table 2 and S1). Interestingly, 32 of these genes were also present in H37Rv (Table 2 and S1). These 32 genes were thus missing in the Nigerian L5 strain exclusively, meaning that its ancestor has genes with a subsequent loss in the Nigerian L5 strain. Those 32 genes – absent in the Nigerian L5 strain only – were sequentially contiguous and formed 3 blocks of 19, 11 and 2 genes (Table S1). We found that 30 of those genes, the blocks of 19 and 11, were separated by 1 gene and represented the L5Nig-Del region (mentioned in the paragraph above, Table 3). Additionally, 11 genes were shared by the 3 PacBio-sequenced genomes of L5 strains, which were absent in H37Rv (Table S2), while 9 genes present in H37Rv were not present in any of the 3 complete (PacBio-sequenced) genomes of L5 strains (Table S3). Two (*Rv2073c*, *Rv2074*) of those nine genes were only present in H37Rv but absent in the complete genomes of L5, L6 and *M. bovis* strains. Note that for the gene presence/absence analysis, the genes are often referred to by the Rv designation but are actually putative orthologues of those H37Rv genes.

**Table 1.** Presence in Illumina-sequenced genomes from 202 L5 strains of genes detected in only 1 of the complete genomes of the 3 L5 strains from Benin, The Gambia and Nigeria

	Gene	Co-ordinates in the specified genome	Functional group	Present in L5 Illumina-sequenced genomes % (*n*=202)
**PcbL5Ben**	PcbL5_01893	2004773–2005918	Cell wall and cell processes	**77.7** (157)
	PcbL5_01894	2006144–2007247	Intermediary metabolism and respiration	**77.7** (157)
	PcbL5_01895	2007448–2010285	Cell wall and cell processes	**77.7** (157)

**Table 2.** Gene content difference between the *M. tuberculosis* H37Rv (L4) genome and the complete (PacBio-sequenced) genomes of three L5 strains from Benin, The Gambia and Nigeria

		Present in			
		**PcbL5Ben**	**PcbL5Gam**	**PcbL5Nig**	**H37Rv**
**Absent in**	**PcbL5Ben**				**9^†^**
	**PcbL5Gam**	2+3*			2+**9^†^**
	**PcbL5Nig**	34+3*	34		32+**9^†^**
	**H37Rv**	**10**‡+3*	**10^‡^**	**10^‡^**	
		4189	4162	4134	4126

*, includes three genes only present in PcbL5Ben.

†, includes nine genes only present in H37Rv.

‡, includes 10 genes shared by the PcbL5 (Benin, The Gambia, Nigeria) and absent in H37Rv.

**Table 3.** Genes in the L5.3.2-Del region (PcbL5Nig-Del) and their function category. None of the 30 genes is an essential gene (Mycobrowser). The 30 genes formed 2 regions: *Rv1493* through *Rv1509* (L5.3.2-Del region 1) and *Rv1511* through *Rv1521* (L5.3.2-Del region 2), separated by the gene *Rv1510*, which is present in the L5.3.2 isolate. All genes in the table are absent from all L5.3.2 genomes (both PacBio- and Illumina-sequenced), except those marked with an asterisk (*), which are present in the PacBio-sequenced genome (PcbL5Nig) but absent in all six Illumina-sequenced genomes, and the one marked with a hash (^#^) (Rv1492), which is a gene present in all L5.3.2 strains and flanking the L5.3.2-specific deletion

Gene name	Size	Co-ordinates in H37Rv	Functional category	Present in L5 Illumina-sequenced genomes % (*n*=202)
Rv1492^#^ (*mutA*)	1848 bp	1 682 157–1 684 004	Lipid metabolism	100 (202)
***Rv1493 (mutB)***	2253 bp	1 684 005–1 686 257	Lipid metabolism	97 (196)
***Rv1494 (mazE4)***	303 bp	1 686 271–1 686 573	Virulence, detoxification, adaptation	97 (196)
***Rv1495 (mazF4)***	318 bp	1 686 570–1 686 887	Virulence, detoxification, adaptation	97 (196)
***Rv1496***	1005 bp	1 686 884–1 687 888	Cell wall and cell processes	97 (196)
***Rv1497 (lipL)***	1290 bp	1 687 941–1 689 230	Intermediary metabolism and respiration	97 (196)
***Rv1498c***	618 bp	1 6893 03–1 689 920	Intermediary metabolism and respiration	97 (196)
***Rv1498A***	213 bp	1 690 134–1 690 346	Conserved hypothetical protein	97 (196)
***Rv1499***	399 bp	16 900 407–1 690 805	Conserved hypothetical protein	97 (196)
***Rv1500***	1029 bp	1 690 850–1 691 878	Intermediary metabolism and respiration	97 (196)
***Rv1501***	822 bp	1 691 890–1 692 711	Conserved hypothetical protein	97 (196)
***Rv1502***	900 bp	1 692 924–1 693 823	Unknown	97 (196)
***Rv1503c***	549 bp	1 693 996–1 694 544	Conserved hypothetical protein	97 (196)
***Rv1504c***	600 bp	1 694 545–1 695 144	Conserved hypothetical protein	97 (196)
***Rv1505c***	666 bp	1 695 281–1 695 946	Conserved hypotheticals	97 (196)
***Rv1506c***	501 bp	1 695 943–1 696 443	Unknown	97 (196)
***Rv1507A***	504 bp	1 697 356–1 697 859	Unknown	97 (196)
***Rv1507c***	696 bp	1 696 727–1 697 422	Conserved hypotheticals	97 (196)
***Rv1508c***	1800 bp	1 698 095–1 699 894	Cell wall and cell processes	97 (196)
***Rv1508A***	636 bp	1 699 866–1 700 228	Conserved hypotheticals	97 (196)
***Rv1509***	882 bp	1 700 212–1 701 093	Unknown	97 (196)
*Rv1510* *	1299 bp	1 701 295–1 702 593	Cell wall and cell processes	97 (196)
***Rv1511 (gmdA)***	1023 bp	1 703 074–1 704 096	Intermediary metabolism and respiration	97 (196)
***Rv1512 (epiA)***	969 bp	1 704 093–1 705 061	Intermediary metabolism and respiration	97 (196)
***Rv1513***	732 bp	1 705 058–1 705 789	Conserved hypothetical protein	97 (196)
***Rv1514c***	789 bp	1 705 807–1 706 595	Conserved hypothetical protein	97 (196)
***Rv1515c***	897 bp	1 706 630–1 707 526	Conserved hypothetical protein	97 (196)
***Rv1516c***	1011 bp	1 707 529–1 708 539	Intermediary metabolism and respiration	97 (196)
***Rv1517***	765 bp	1 708 871–1 709 635	Cell wall and cell processes	97 (196)
***Rv1518***	960 bp	1 709 644–1 710 603	Conserved hypothetical protein	97 (196)
***Rv1519***	270 bp	1 710 733–1 711 002	Conserved hypothetical protein	97 (196)
***Rv1520***	1041 bp	1 711 028–1 712 068	Intermediary metabolism and respiration	97 (196)
***Rv1521 (fadD25)***	1752 bp	1 712 302–1 714 053	Lipid metabolism	97 (196)
Rv1522c* (mmpL12)	3441 bp	1 714 172–1 717 612	Cell wall and cell processes	97 (196)

Six of the genes shared by the three complete (PacBio-sequenced) genomes of L5 strains were confirmed pseudogenes in H37Rv (Table S4). Three genes present in H37Rv were confirmed to be pseudogenes in the three complete (PacBio-sequenced) genomes of the L5 strains (Table S5).

### Gene presence/absence and related SNPs in lineage-specific genes in a wider set of clinical strains

The mapping estimates of the Illumina-sequenced genomes are presented in Table 4. Three (ERR502505, ERR751302, ERR1215478) of the 205 Illumina-sequenced genomes were identified as mixed infection strains based on their MTBseq output, and thus excluded from the analysis. Mapping quality and coverage against a complete L5 reference was superior to the H37Rv reference approach (Table 3, Fig. S1), as expected. Using the complete (PacBio-sequenced) genomes from Benin and The Gambia strains, yielded similar mapping estimates that were better than those of the genome of the Nigerian L5 strain, likely due to the large deletion in this genome. The genes specific to PcbL5Ben [3, region PcbL5Ben_1893 through PcbL5Ben_1895; none specific to PcbL5Gam (0), PcbL5Nig (0)] were each found in 77.7 % (157/202, Table 1) of the 202 Illumina-sequenced genomes of L5 strains.

**Table 4.** Mapping of Illumina-sequenced genomes of the 202 L5 strains to the *M. tuberculosis* H37Rv (L4) genome and complete genomes of 3 L5 strains from Benin, The Gambia and Nigeria (mapping statistics/estimates). The best mapping results (numbers) are written in bold. When the best mapping result has been obtained for PcbL5Nig as the reference, the next best result is also written in bold (as PcbL5Nig compared to the other 2 PcbL5 genomes missed a 30-gene region)

	H37Rv	PcbL5Ben	PcbL5Gam	PcbL5Nig
Reads				
**Mean of percentage L5 reads mapped to**	96.9	**97.5**	97.3	96.5
**Mean of unambiguous coverage mean**	122.3	123.2	**123.3**	123.0
Bases				
**Mean of percentage unambiguous total bases**	98.0	98.8	**98.9**	98.2
**Mean uncovered**	30 599.0	18 916.7	14 766.4	35 340.4
**Mean SNP**	2209.7	529.5	**513.0**	**503.3**
**Mean deletions**	374.1	96.5	**96.4**	97.9
**Mean insertions**	239.5	**77.0**	107.6	**60.8**
**Mean substitutions (including stop codons)**	1193	**0**	**0**	**0**
Genes				
**Mean of percentage gene mapped (presence)**	99.59	99.71	**99.79**	99.76
**L5 illumina having all the ref. genome genes, % (** ***n*** **=202)**	0	2 (4)	7.4 (15)	0
**Mean gene count difference between L5 Illumina-sequenced genomes (gene count per Illumina-sequenced genome minus minimum gene count)**	30.3	**38.2**	34.4	32.8

**Fig. 1.**
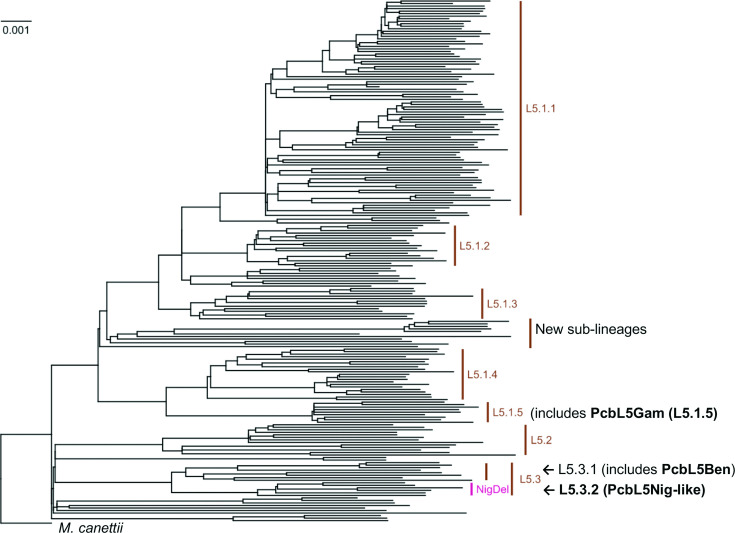
Phylogenetic tree showing the Illumina-sequenced genomes of the six L5 strains (L5.3.2) similar to the complete (PacBio-sequenced) genome of the Nigerian L5 strain (PcbL5Nig) and the position of the other two PacBio-sequenced L5 genomes (PcbL5Ben and PcbL5Gam). NigDel=L5Nig-Del=L5.3.2-Del=region of 30 genes (2 blocks of 19 and 11 genes: *Rv1493* through *Rv1509* and *Rv1511* through *Rv1521*) missing in L5.3.2 strains but present in all other L5 strains (L5.1, L5.2, L5.3.1 and new sub-lineages).

Interestingly, 3 % of the Illumina-sequenced genomes of L5 strains (6/202) had similar patterns of large gene loss like the Nigerian complete (PacBio-sequenced) genome, as they missed 30 of the 32 genes present in the genomes of the Benin and Gambian strains and H37Rv. These six PcbL5Nig-like Illumina-sequenced genomes of L5 strains formed a monophyletic group within the L5 clade (Fig. 1), suggesting a single loss of these gene clusters, although those six L5 strains genomes originated from several different countries, including Benin, Ghana and Nigeria. The 2 blocks of 19 (15 984 bp, *Rv1493* through *Rv1509*) and 11 genes (10 209 bp, *Rv1511* through *Rv1521*), separated by 1 gene (3441 bp, *Rv1510*), amounted in total to 26 193 bp. The two blocks contain genes whose annotations include *mutB*, *mazE4*, *mazF4* and others with various putative functions (Table 3, Table S6); none of them were essential genes (Mycobrowser).

Four of the 11 genes present in the 3 complete (PacBio-sequenced) genomes of the L5 strains but absent in H37Rv were found in all 202 Illumina-sequenced genomes of the L5 strains (Table 5), while the others were found in variable amounts [80.2–98 % (162–198)] (Table S2). These four genes include: *Mb2048c (*of unknown function, belonging to the *RvD1* deletion in H37Rv*)*, a PE/PPE gene, a hypothetical protein (possibly an IS*256* transposase) and a hypothetical protein possibly related to the CAAX conserved domain (Table 5). Two of these four genes were only present in L5 genomes (a PE/PPE gene and a hypothetical protein, possibly CAAX conserved domain), while the remaining two [*Mb2048c (RvD1)* and hypothetical protein, possibly IS256 transposase] were found in L6 and *M. bovis* as well (Tables 5 and S2). Importantly for phylogenetic purposes, SNPs were detected in all 4 genes in 1.5–3.5 % of the 202 Illumina-sequenced genomes of L5 strains (Table S2). Predicted functions for all L5-specific genes are listed in Table S2.

**Table 5.** Genes present or absent in all L5 genomes compared to H37Rv. The genes are ordered in the table along with their flanking genes, as in the genome. The RD regions are those reported by Gordon *et al.* [13]

Gene name	Present in	Size	Co-ordinates in H37Rv	Co-ordinates in PcbL5Ben	Functional category	Present in L5 Illumina-sequenced genomes % (*n*=202)	Belongs to RD# (no. total of genes forming the RD)
PcbL5Ben_2128 (*Rv1976c*)			2218844–2219251	2244287–2244565	Conserved hypothetical protein	100 (202)	RD7
**PcbL5Ben_2129**	**L5**	648 bp	–	2244695–2245342	**PE/PPE**	**100** (202)	
**PcbL5Ben-2130**	**L5**	600 bp	–	2246122–2246721	**Hypothetical protein**	**100** (202)	
**Rv1977**	**H37Rv, L6, *M. bovis***	1047 bp	2219754–2220800	–	**Conserved hypothetical protein**	**0** (0)	**RD7 (14 genes)**
Rv1978	H37Rv, L6, *M. bovis*	849 bp	2220908–2221756	–	Conserved hypothetical protein	1 (2)	RD2
**Rv1979c**	**H37Rv, L6, *M. bovis***	1446 bp	2221719–2223164	–	**Cell wall and cell processes**	**0** (0)	**RD2 (10 genes)**
PcbL5Ben_2131 (*Rv1980* (*mpt64*))			2223343–2224029	2246867–2247553	Cell wall and cell processes	100 (202)	RD2
………//………							
PcbL5Ben_2149 (Rv1992 (*ctpG*))			2234991–2237306	2258516–2259319	Cell wall and cell processes	100 (202)	
**Rv1993c**	**H37Rv, L6, *M. bovis***	273 bp	2237303–2237575	–	**Conserved hypothetical protein**	**0** (0)	
Rv1994c (ctmR)	H37Rv, L6, *M. bovis*	357 bp	2237628–2237984	–	Regulatory proteins	0.5 (1)	
**Rv1995**	**H37Rv, L6, *M. bovis***	729 bp	2238141–2238908	–	**Conserved hypothetical protein**	**0** (0)	
PcbL5Ben_2150 (Rv1996)			2239004–2239957	2259333–2260169	Virulence, detoxification, adaptation	100 (202)	
………//………							
PcbL5Ben_2180 (Rv2023A)			2268268–2268726	2289843–2290475	Conserved hypothetical protein	100 (202)	
**PcbL5Ben-2181**	**L5, L6, *M. bovis***	237 bp	–	2290472–2290708	Hypothetical protein	**100** (202)	
**PcbL5Ben-2182 (Mb2048c, RvD1)**	**L5, L6, *M. bovis***	897 bp	–	2290915–2291811	Unknown function	**100** (202)	
PcbL5Ben_2183 (Rv2024c)			2268693–2270240	2291995–2296815	Conserved hypothetical protein	100 (202)	
………//………							
PcbL5Ben_2231, PcbL5Ben_2232 (Rv2072 (cobL))			2328974–2330146	2355547–2356422, 2356373–2356561	Intermediary metabolism and respiration	100 (202)	RD9 (4 genes, including Rv2072 truncated)
**Rv2073c**	**H37Rv**	750 bp	2330214–2330963	–	**Intermediary metabolism and respiration**	**0** (0)	**RD9**
**Rv2074**	**H37Rv**	408 bp	2330993–2331406		**Intermediary metabolism and respiration**	**0** (0)	**RD9**
PcbL5Ben_2233 (Rv2075c)			2331416–2332879	2356636–2357388	Cell wall and cell processes	100 (202)	RD9 (4 genes, including Rv2075c truncated)

Six (*Rv1977, Rv1979c*, *Rv1993c*, *Rv1995*, *Rv2073c, Rv2074*) of the 9 genes that were present in H37Rv and absent in the 3 complete (PacBio-sequenced) genomes of L5 strains were absent in all 202 Illumina-sequenced genomes of L5 strains (Table 5 and S3). *Rv1977* is a conserved hypothetical, probably a peptidase; *Rv1979c* is involved in ‘cell wall and cell processes’, probably a permease; *Rv1993c* and *Rv1995* are conserved hypotheticals; and *Rv2073c* and *Rv2074* are involved in ‘intermediary metabolism and respiration’, with *Rv2073c* probably a dehydrogenase and *Rv2074* a pyridoxamine-5-phosphate oxidase (Mycobrowser, Table 5). Four of these genes (*Rv1977*, *Rv1979c*, *Rv1993c*, *Rv1995*) were absent (did not have orthologues) in L5 strains only (i.e. present in L6 and *M. bovis* reference genomes), while the other two (*Rv2073c* and *Rv2074*) were also absent in L6 and *M. bovis* (Table 5). The three other genes absent from the complete (PacBio-sequenced) genomes were present in a minority of the L5 Illumina-sequenced genomes (Table S3, Fig. S1). Predicted functions for all nine genes are listed in Table S3.

### Sub-lineage of the strains (PacBio and Illumina-sequenced genomes)

The determination of the strains sub-lineage revealed that the PcbL5Ben strain is an L5.3 strain that contains the 30 genes (2 blocks of 19 and 11 genes: *Rv1493* through *Rv1509* and *Rv1511* through *Rv1521*), whereas the PcbL5Nig strain is also a L5.3 strain, but placed in a different clade (Fig. 1). Based on this new RD (L5Nig-Del), we classified PcbL5Ben as a L5.3.1 strain, the PcbL5Nig as a L5.3.2 strain. The PcbL5Gam strain is a L5.1.5 strain. The distribution of the Illumina-sequenced genomes is outlined in Table S7, amounting to a total of 146 L5.1 strains (72.3 %); 13 (6.4 %) L5.2 strains; 14 L5.3 strains (6.9 %); and 29 (14.4 %) strains of unknown (potentially new) sub-lineages.

### Impact of the reference genome selection on genetic distances used for transmission clustering rates

Transmission analysis was undertaken on an expanded set of 352 L5 strains using each of the four reference genomes (H37Rv, PcbL5Ben, PcbL5Gam and PcbL5Nig) for the SNP mapping. The current gold standard is to use the H37Rv genome (NC_000962.3) for calling SNPs and then creating transmission clusters at a specific SNP cut-off differentiating two and more strains. For this dataset, using a 12 SNP cut-off, it was found that 40.6 % (*n*=144) of strains were within a transmission cluster with at least 1 other isolate (Table 6), for a total of 55 clusters. Using the PcbL5Ben genome instead of the H37Rv genome as the reference reduced the number of clusters to 54, resulting in a clustering rate of 39.7 % at a 12 SNP cut-off (Table 6). A similar reduction in transmission clustering was observed when using the PcbL5Nig genome as a reference but not when using the PcbL5Gam (Table 6). When a more conservative SNP cut-off was used, such as one SNP or five SNPs, the reduction in transmission clustering rates was more pronounced and was observed for all the PcbL5 genomes (Table 6). This demonstrates that even with this small dataset, transmission cluster estimations are affected by the selection of the reference genome, with potential overestimation of recent transmission when using H37Rv as the reference genome.

**Table 6.** Comparison of transmission clustering rates based on choice of reference genome. Short-read data from 355 L5 strains were mapped against each of the 4 reference genomes for SNP calling. Distance matrices between all strains were constructed per the reference approach and transmission clusters were defined based on specific SNP cut-offs

1 SNP			
**Reference used**	**In transmission cluster**	**Percentage of total dataset**	**No. of clusters**
H37Rv	100	28.17	44
PcbL5Ben	94	26.48	43
PcbL5Gam	95	26.76	43
PcbL5Nig	95	26.76	43
**5 SNP**			
**Reference used**	**In transmission cluster**	**Percentage of total dataset**	**No. of clusters**
H37Rv	129	36.34	53
PcbL5Ben	124	34.93	51
PcbL5Gam	124	34.93	51
PcbL5Nig	124	34.93	51
**12 SNP**			
**Reference used**	**In transmission cluster**	**Percentage of total dataset**	**No. of clusters**
H37Rv	144	40.56	55
PcbL5Ben	141	39.72	54
PcbL5Gam	144	40.56	55
PcbL5Nig	141	39.72	54

## Discussion

Comparison of complete (PacBio-sequenced) genomes and Illumina-sequenced genomes from clinical strains revealed gene content diversity both within L5 strains and between L5 strains and strains of other lineages. This diversity also had an impact on clinical epidemiology analysis of L5 strains, with the choice of reference genome affecting the estimation of recent transmission.

Several genes were found to be absent from the genome of either L5 strains only or also from strains of the closely related lineages L6 and *M. bovis*. Most of these genes are located within RDs previously described as being absent in one or all of these lineages.

RD9, consisting of *Rv2073c* and *Rv2074* and partial *Rv2072* and *Rv2075c* [13], has previously been reported to be absent in L5, L6 and *M. bovis* [10, 12, 15]. In this study, we confirmed that *Rv2073c* and *Rv2074* are absent in strains of these lineages. *Rv2072* and *Rv2075c* were present in their truncated form in the genomes of all L5 strains as previously described [13]. *Rv2073c* is an NAD(P)-dependent oxidoreductase (NCBI) that catalyzes a wide range of reactions and is involved in redox sensor mechanisms [64, 65]. *Rv2074* was previously thought to be a pyridoxine (vitamin B6) oxidase, but is now known to be a F420-dependent biliverdin reductase, a cofactor of vitamin B6 synthesis [66, 67]. Vitamin B6 is essential for the survival and virulence of *M. tuberculosis* [68] and its cofactor (F420-dependent biliverdin reductase) is implicated in immune-evasive mechanisms to allow bacterial persistence [66, 67] (Table S3). Recently, it has been reported that the synthesis of F420 might be stimulated by phosphoenolpyruvate [69], which is the precursor to pyruvate, a supplement often added to culture medium to improve the *in vitro* growth of L5, L6 and *M. bovis* strains. Furthermore, there is a suggestion that F420 is needed for the activation of the antituberculosis drugs pretomanid and delamanid [69].

Another gene absent in L5 strains is *Rv1977*. This gene is one of the 14 genes contained in RD7 [13]), which is known to be lacking completely from strains of L6 and animal-adapted lineages such as *M. bovis* [12]. Similarly, *Rv1979c*, 1 of 10 genes that make up RD2, was absent from genomes of L5 strains; this RD is also absent from late-generation *M. bovis* BCG such as *M. bovis* BCG Pasteur strains [12]*. Rv1978,* another of the 10 genes in RD2, and located in the region *Rv1977-Rv1978-Rv1979c* was absent in the 3 complete (PacBio-sequenced) genomes of L5 strains, but present in 2 L5 strains Illumina-sequenced genomes [2/202, 1 %; further confirmed using blast search and manual check of the gene in the reads of the 2 L5 strains genomes (ERR439931 and ERR4192386)], making up the region containing *Rv1977*, *Rv1978* and *Rv1979c*, a region absent in most of L5 strains (99 %, 200/202). *Rv1979c* has been associated with clofazimine and bedaquiline resistance [70, 71], two of the drugs used for the treatment of rifampicin- and multidrug-resistant TB [72, 73], but minimal inhibitory concentration testing of clofazimine in five L5 strains did not find that the deletion conferred resistance [74]. Further studies and protein function discovery is needed to investigate the consequences of the absence of those genes in genomes of L5 strains. *Rv1978* is required for bacterial survival in macrophages [75].

*Rv1993c* and *Rv1995* were absent in all L5 genomes. *Rv1994c* a gene outside of known RDs, located in the region *Rv1993c-Rv1994-Rv1994* was absent in the three complete (PacBio-sequenced) genomes of L5 strains, yet present in one L5 strain Illumina-sequenced genome [1/202, 0.5 %; further confirmed, using blast search and manual checking of *Rv1994* in the reads of the concerned L5 strain genome (ERR439931)]. This resulted in the absence, in most of the L5 strains (99.5 %, 201/202) of the region containing *Rv1993c*, *Rv1994c* and *Rv1995. Rv1994c* is involved in the regulation and transport (efflux) of toxic metals, especially copper, which is toxic in excess and may hamper *in vitro* growth [76–80] (Table S3). *Rv1993c* forms with *Rv1994* (*cmtR*) the operon *cmtR-Rv1993c-ctpG*, and *Rv1995* is involved in oxygen transport (Table S3).

In combination, the absence of these genes in L5 strains suggests that they would be less likely to survive in macrophages (*Rv1978*), have reduced growth *in vitro* (*Rv1994c*, as previously found by [27]), and be less immune-evasive, less persistent and less virulent (*Rv2074*) than L4 strains (at least H37Rv), as previously suggested for L5 and L6 strains [5, 32]). In addition, the absence of *Rv2074* in most L5 strains (and in the complete genomes of L6 and *M. bovis* as well) suggests that L6 and *M. bovis* strains would also be less likely to be immune-evasive, to be persistent and to survive than L4 strains. Despite the presence of vitamin B6 in boiled egg (thus Lowenstein–Jensen medium), the quantity of vitamin B6 in the Lowenstein–Jensen medium is probably insufficient to allow a high yield of growth of L5, L6 and *M. bovis* strains in culture (survival). Further studies, including vitamin B6 supplementation, could investigate the consequences of the absence of those genes in L5 strains (and L6 and *M. bovis* for *Rv2074*).

In contrast to the absence of some genes in RD2 and RD7, or all genes in RD9, RD5 was found to be present in its entirety in nearly all sequenced genomes of L5 strains. Some authors reported that up to 45 % of L5 strains missed RD5, while others reported that RD5 is present in most L5 strains [35, 81]. The RD5 region includes *Rv2346* (truncated), *Rv2347*, *Rv2348*, *Rv2349c* (*plcC*), *Rv2350c* (*plcB*), *Rv2351c* (*plcA*) and a PPE gene [13]. Our findings showed that of the 202 Illumina-sequenced L5 strains, the majority (196, 97 %) had the RD5 region in its entirety, whereas 6 L5 strains (3 %) missed a part (one or 2 genes among the non-PPE genes) of the RD5 region. Likewise, in contrast to a previous report [10], our data did not confirm that all L5 strains lack RD711 (coordinates: 1 501 713–1 503 655 in H37Rv [82]) composed of *Rv1333* (truncated)*, Rv1334, Rv1335* and *Rv1336* (truncated). The lacking of RD711 was rather observed in all L5.1 strains in our dataset, which is in agreement with the report of Ates *et al.* [35] and also observed among 58.6 %(17/29) of L5 strains of unknown sub-lineages (Table S7).

We found that four genes, present in all of the L5 strains in our multi-country collection, were absent (no orthologues) in the H37Rv genome. Those genes represent 2382 bp, which is around 0.05 % of the total length (base pair) of a typical genome of an MTBC strain. Our findings are in line with those of other reports indicating that some genes present in genomes of clinical MTBC strains were absent (no orthologues) in the H37Rv genome [46, 47]. Although these gene differences are small, they did affect the distance matrices used for transmission clustering analyses (Table 6). Overall, H37Rv-based mapping was found to place a slightly higher percentage of strains in transmission clusters than any L5-based mapping approach, especially at lower SNP cut-offs. This has implications for molecular epidemiology in West Africa, where most L5 strains are found. Additionally, if similar scenarios exist for other lineages, this may result in changes to all non-L4 transmission analyses.

Our findings support past recommendations to use additional reference genomes that are different from H37Rv [46, 83, 84]. Although another study [85] concluded that there is no need to use a lineage-specific reference genome, their observation was only based on the analysis of L4 clinical strains and focused on SNPs and short indels, not larger gene deletions or SNPs within these regions. In contrast, our findings indicate that mapping of NGS data from L5 strains to L4 reference will have an impact in terms of both reference genome coverage and coverage of lineage-specific genes.

While the use of a single L5 genome as reference would have many benefits over H37Rv for particular study questions, several gene content differences were still observed within the L5 lineage. One (PcbL5Ben) of the three genomes of L5 strains sequenced with PacBio had genes that were not shared with the two others that were all found in Illumina-sequenced genomes of L5 strains (thus excluding exogenous contamination) (Table 1). The L5NigDel was also found in the genomes of six L5 strains from different countries. These six strains formed a monophyletic group of the (SNP-defined) L5.3 sub-lineage [6] (Fig. 1), suggesting that the loss of these 30 genes is a marker for strains of L5.3.2 sub-lineage, compared to the sub-lineage L5.3.1 strains that have these genes intact. Indeed, deletions, as described for MTBC lineages [10, 45, 48], may also be limited to sub-lineages.

The between- and within-lineage differences in both gene content and potential functionality indicate a need for more closed genomes of MTBC sub-lineages to be constructed. Due to the impact of reference choice on transmission studies, there is now a need for varying approaches to NGS data analysis to be considered. For instance, sub-lineage-specific reference genomes could improve resolution, although such ad hoc (e.g. outbreak-specific) reference genomes are only specific to that particular situation/outbreak/population/lineage and cannot be used in another context, making comparisons between lineages and settings difficult. Alternatively, a pangenome-based reference genome(s) capturing all the known diversity may be required instead. This can take two forms: a composite genome containing all the genes found in strains of all lineages and sub-lineages of the MTBC (i.e. both the core and accessory genome) [86], represented as a graph instead of a single sequence [87–92], or a selection of reference genomes, with mapping to all or a subset undertaken, as has been done with strains of *Mycobacterium chimaera* and other pathogens [93, 94]. Other authors have also reported that, because of the genetic variability between strains, using a single strain genome as reference genome lacks accuracy [95–97]. This MTBC-wide pangenome approach has been suggested before, including for other organisms [38, 47, 95–100]. However, such an approach also has its own drawbacks, including difficulty in mapping reads that bridge the boundaries between accessory genes and the rest of the genome, comparing strains of different lineages including phylogenetic analyses and retention of gene names and codes in clinical use, where H37Rv is deeply embedded [89]. The specific sequence of each orthologue gene would also need to be chosen for such a reference, with the inferred ancestral genome representative of MTBC lineages approach being the most likely method [22, 42, 101].

Another alternative approach is a *de novo* assembly reference-free approach [38, 96, 102, 103], where strains are either assembled into contigs without the use of a reference genome or compared to each other without first calling SNPs. This would allow for clustering of samples regardless of lineage, e.g. for genotyping or transmission analyses using Mash (software for fast/meta -genome distance estimation technique) [104] but would require new cut-offs for defining clusters and many additional steps for further gene annotation and between-sample comparisons, making its clinical use potentially confusing.

A limitation of this study is that the complete (PacBio) and short-read (Illumina) genomes were derived from positive cultures, excluding possible minority L5 strain diversity, as L5 strains are overrepresented in negative cultures [27]. WGS applied directly to sputum is increasingly needed, especially for ancestral lineages (including L5 and L6), where negative culture or dysgonic isolates are more common and are a challenge for DNA extraction [26, 27]. There is also a limitation regarding the comparison of gene presence/absence in strains of L5 versus L4 (including H37Rv), L6, *M. bovis* complete genomes; which is that our study only included a single complete genome of one strain of L4, L6 and *M. bovis*, respectively, while strains of these lineages may display similar variability to the intra-L5 variability we observed in our study. Of note, the genes that are present in L5 strains but not specific to L5 strains could be either active or inactive (mutated), requiring additional *in vitro* or *in vivo* validations to fully elucidate the metabolic profile of these strains.

In conclusion, the use of a (sub-)lineage reference genome can increase resolution for strain comparison in comparison to a H37Rv-based mapping approach for L5 genome analyses for epidemiology (transmission), phylogeny and sub-lineage determination. Still, the use of a (sub-)lineage reference genome may miss some within-lineage gene differences. For drug resistance detection in clinical L5 strains or strains of other lineages, H37Rv could still be used as a reference genome as resistance-related mutations are usually among the core genes (shared across all lineages). The high within-lineage gene content variability suggests that the pangenome of MTBC strains may be larger (at least by 5092 bp) than previously thought, implying that a reference-free genome assembly (*de novo assembly*) approach may be needed.

## Supplementary Data

Supplementary material 1
