# Supplementary material for: Mycobacterium tuberculosis complex lineage 5 exhibits high levels of within-lineage genomic diversity and differing gene content compared to the type strain H37Rv
Source: Microb Genom. 2021 Jul 9;7(7):000437. doi: 10.1099/mgen.0.000437 (PMC8477398; doi:10.1099/mgen.0.000437)
Supplement: Supplementary material 1 [file mgen-7-0437-s001.pdf]

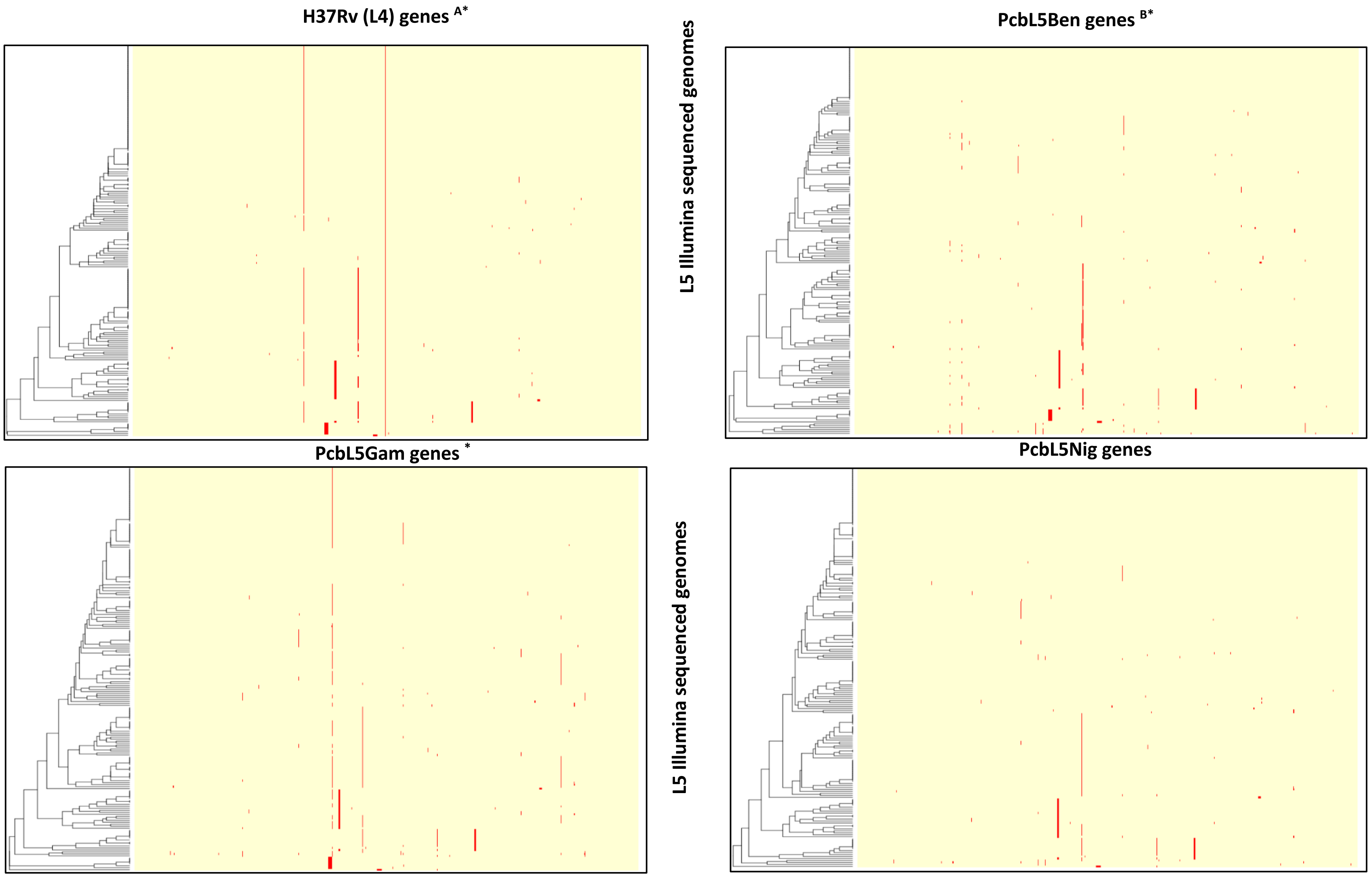

Figure S1. Mapping of 202 clinical (Illumina platform sequenced) genomes (L5_ig) of *M. africanum* L5 from various countries to *M. tuberculosis* H37Rv (L4) and three *M. africanum* L5 isolates (completed PacBio sequenced genomes) from Benin, The Gambia and Nigeria as reference (PcbL5Ben; PcbL5Gam; PcbL5Nig).

(A) **6**/9 genes present in H37Rv but absent in the three L5_cg were **absent in all 202 L5_ig.**
(B) **3**/3 genes **present in PcbL5Ben but absent in the two other L5 completed PacBio sequenced genomes** were **present in 157/202 L5_ig.**

* The 32 genes absent in PcbL5Nig but present in PcbL5Ben, PcbL5Gam and *H37Rv* were also absent in 6/202 L5_ig (Fig.1).

.... or .... gene absent

Table S1. Gene difference within the three L5 completed PacBio sequenced genomes from Benin, Nigeria or The Gambia, and between these three genomes and *M. tuberculosis* H37Rv

Genes	Present in PcbL5Ben	Present in PcbL5Gam	Present in PcbL5Nig	Present in H37Rv
**Unshared (present in that genome only)**	PcbL5Ben_01893, PcbL5Ben_01894, PcbL5Ben_01895, PcbL5Ben_02043, PcbL5Ben_03043	PcbL5Gam_02028, PcbL5Gam_03020	PcbL5Nig_02001	H37Rv_02002, H37Rv_02085, H37Rv_02086, H37Rv_02087, H37Rv_02107, H37Rv_02108, H37Rv_02109, H37Rv_02188, H37Rv_02189
**Absent in PcbL5Ben**		PcbL5Gam_unshared	PcbL5Nig_unshared	H37Rv_unshared
**Suspected pseudogenes in PcbL5Ben**		PcbL5Gam_00036, PcbL5Gam_00955, PcbL5Gam_02045, PcbL5Gam_03308, PcbL5Gam_03376	PcbL5Nig_00011, PcbL5Nig_00036, PcbL5Nig_00953, PcbL5Nig_01794, PcbL5Nig_01853, PcbL5Nig_01896, PcbL5Nig_02018, PcbL5Nig_02119, PcbL5Nig_02291, PcbL5Nig_02467	H37Rv_00012, H37Rv_00036, H37Rv_00528, H37Rv_01371, H37Rv_01801, H37Rv_02020, H37Rv_02463, H37Rv_02556, H37Rv_02951, H37Rv_03349, H37Rv_03797
**Absent in PcbL5Gam**	PcbL5Ben_01443, PcbL5Ben_01444, PcbL5Ben_unshared	PcbL5Gam_unshared	PcbL5Nig_unshared	H37Rv_01413, H37Rv_01414, H37Rv_unshared
**Suspected pseudogenes in PcbL5Gam**	PcbL5Ben_01064, PcbL5Ben_02378, PcbL5Ben_02486, PcbL5Ben_03113		PcbL5Nig_00011, PcbL5Nig_01794, PcbL5Nig_01853, PcbL5Nig_01896, PcbL5Nig_02119, PcbL5Nig_02291, PcbL5Nig_02467	H37Rv_00012, H37Rv_00528, H37Rv_01371, H37Rv_01801, H37Rv_02329, H37Rv_02463, H37Rv_02556, H37Rv_02951, H37Rv_03797,
**Absent* in PcbL5Nig**	PcbL5Ben_01616, PcbL5Ben_01617, PcbL5Ben_01618, PcbL5Ben_01619, PcbL5Ben_01620, PcbL5Ben_01621, PcbL5Ben_01622, PcbL5Ben_01623, PcbL5Ben_01624, PcbL5Ben_01625, PcbL5Ben_01626, PcbL5Ben_01627,	PcbL5Gam_01603, PcbL5Gam_01604, PcbL5Gam_01605, PcbL5Gam_01606, PcbL5Gam_01607, PcbL5Gam_01608, pcbL5Gam_01609, PcbL5Gam_01610, PcbL5Gam_01611, PcbL5Gam_01612, pcbL5Gam_01613, PcbL5Gam_01614,		H37Rv_01580 H37Rv_01581, H37Rv_01582, H37Rv_01583, H37Rv_01584, H37Rv_01585, H37Rv_01586, H37Rv_01587, H37Rv_01588, H37Rv_01589, H37Rv_01590, H37Rv_01591,

	PcbL5Ben_01628,<br>PcbL5Ben_01629,<br>PcbL5Ben_01630,<br>PcbL5Ben_01631,<br>PcbL5Ben_01632,<br>PcbL5Ben_01633,<br>PcbL5Ben_01634,<br><br>PcbL5Ben_01636,<br>PcbL5Ben_01637,<br>PcbL5Ben_01638,<br>PcbL5Ben_01639,<br>PcbL5Ben_01640,<br>PcbL5Ben_01641,<br>PcbL5Ben_01642,<br>PcbL5Ben_01643,<br>PcbL5Ben_01644,<br>PcbL5Ben_01645,<br>PcbL5Ben_01646<br><br>PcbL5Ben_01891,<br>PcbL5Ben_01892,<br>PcbL5Ben_02248,<br>PcbL5Ben_unshared	pcbL5Gam_01615,<br>PcbL5Gam_01616,<br>PcbL5Gam_01617,<br>PcbL5Gam_01618,<br>pcbL5Gam_01619,<br>PcbL5Gam_01620,<br>PcbL5Gam_01621,<br><br>PcbL5Gam_01623,<br>PcbL5Gam_01624,<br>PcbL5Gam_01625,<br>pcbL5Gam_01626,<br>PcbL5Gam_01627,<br>PcbL5Gam_01628,<br>PcbL5Gam_01629,<br>PcbL5Gam_01630,<br>PcbL5Gam_01631,<br>PcbL5Gam_01632,<br>pcbL5Gam_01633<br><br>PcbL5Gam_01881,<br>PcbL5Gam_01882,<br>PcbL5Gam_02228,<br>PcbL5Gam_unshared		H37Rv_01592,<br>H37Rv_01593,<br>H37Rv_01594,<br>H37Rv_01595,<br>H37Rv_01596,<br>H37Rv_01597,<br>H37Rv_01598,<br><br>H37Rv_01600,<br>H37Rv_01601,<br>H37Rv_01602,<br>H37Rv_01603,<br>H37Rv_01604,<br>H37Rv_01605,<br>H37Rv_01606,<br>H37Rv_01607,<br>H37Rv_01608,<br>H37Rv_01609,<br>H37Rv_01610,<br><br>H37Rv_01853,<br>H37Rv_01854,<br>H37Rv_02202<br>H37Rv_unshared
**Suspected pseudogenes in PcbL5Nig**	PcbL5Ben_01064,<br>PcbL5Ben_01718,<br>PcbL5Ben_02486	PcbL5Gam_01705,<br>PcbL5Gam_03308,<br>PcbL5Gam_03376		H37Rv_00528,<br>H37Rv_01371,<br>H37Rv_01679,<br>H37Rv_02463,<br>H37Rv_02556,<br>H37Rv_02951,<br>H37Rv_03349,<br>H37Rv_03797
**Absent in H37Rv**	PcbL5Ben_01364,<br>PcbL5Ben_01365,<br>PcbL5Ben_02129,<br>PcbL5Ben_02130,<br>PcbL5Ben_02043,<br>PcbL5Ben_02181,<br>PcbL5Ben_02182,<br>PcbL5Ben_03555,<br>PcbL5Ben_03556,<br>PcbL5Ben_03557,<br>PcbL5Ben_03669<br>PcbL5Ben_unshared	PcbL5Gam_01359,<br>PcbL5Gam_01360,<br>PcbL5Gam_02112,<br>PcbL5Gam_02113,<br>PcbL5Gam_02028,<br>PcbL5Gam_02163,<br>PcbL5Gam_02164,<br>PcbL5Gam_03527,<br>PcbL5Gam_03528,<br>PcbL5Gam_03529,<br>PcbL5Gam_03643,<br>PcbL5Gam_unshared	PcbL5Nig_01357,<br>PcbL5Nig_01358,<br>PcbL5Nig_02085,<br>PcbL5Nig_02086,<br>PcbL5Nig_02001,<br>PcbL5Nig_02137,<br>PcbL5Nig_02138,<br>PcbL5Nig_03500,<br>PcbL5Nig_03503,<br>PcbL5Nig_03504,<br>PcbL5Nig_03616,<br>PcbL5Nig_unshared	
**Suspected pseudogenes in H37Rv**	PcbL5Ben_00749,<br>PcbL5Ben_00828,<br>PcbL5Ben_01064,<br>PcbL5Ben_01108,<br>PcbL5Ben_02128,<br>PcbL5Ben_02486,	PcbL5Gam_00750,<br>PcbL5Gam_00826,<br>PcbL5Gam_00955,<br>PcbL5Gam_01103,<br>PcbL5Gam_01879,<br>PcbL5Gam_02111,	PcbL5Nig_00748,<br>PcbL5Nig_00824,<br>PcbL5Nig_00953,<br>PcbL5Nig_01100,<br>PcbL5Nig_01846,<br>PcbL5Nig_01853,	

	PcbL5Ben_03113, PcbL5Ben_03141, PcbL5Ben_03667	PcbL5Gam_03117, PcbL5Gam_03308, PcbL5Gam_03641	PcbL5Nig_01896, PcbL5Nig_02084, PcbL5Nig_02119, PcbL5Nig_02291, PcbL5Nig_02467, PcbL5Nig_03064, PcbL5Nig_03092, PcbL5Nig_03614	

*Genes highlighted in the same color are contiguous and form a block.

Table S2. Presence /absence in 202 L5 isolates Illumina sequenced genomes of the 11 genes shared by the three L5 completed genomes but absent in the *M. tuberculosis* H37Rv genome. The 4 genes present in all L5 strains but absent in H37Rv are presented in the grey-background lines of the table.

Genes present in L5 completed PacBio sequenced genomes but absent in H37Rv	Length (bp)	Present in L5 isolates Illumina sequenced genome n=202 (%)	SNP in L5-specific genes			Gene function			Present in complete genome of
			PcbL5Ben as ref n=202 (%)	PcbL5Gam as ref n=202 (%)	PcbL5Nig as ref n=202 (%)	Functional group (Mycobrowser)	genes	Summary of role based on the literature	
**PcbL5Ben_1364**	546 bp	188 (93.1)				ABC transporter ATP-binding protein/permease (Prokka)	Transmembrane ATP-binding protein ABC transporter	Conserved domains: FHA and CcmA (NCBI)<br>FHA (forkhead associated domain, COG1716 (T)) binds pSer, pThr, pTyr (Signal transduction mechanisms)<br>CcmA (COG1131 (V)), ABC-type multidrug transport system, ATPase component (Defense mechanisms (NCBI))	L5, bovis
**PcbL5Ben_1365**	1902 bp	188 (93.1)				Same as PcbL5Ben_1364	Same as PcbL5Ben_1364	Same as PcbL5Ben_1364	L5, bovis
**PcbL5Ben_2043**	150 bp	192 (95)				Hypothetical protein	Hypothetical protein		
**PcbL5Ben_2129#**	648 bp	**202 (100)**	4 (2)	4 (2)	202* (100)	**PE/PPE**	PPE		**Only L5**
**PcbL5Ben_2130#**	600 bp	**202 (100)**	7 (3.5)	7 (3.5)	7 (3.5)	Not in Mycobrowser	**Hypothetical protein possibly CAAX conserved domain** (NCBI)	Involved in post-translation modification by attaching to the isoprenoid proteins in the process called prenylation (NCBI). Most of CAAX box proteins do not have a transmembrane domain, thus, the prenylation process is crucial for the function of many signal transduction proteins (Gao, Liao, and Yang 2009)	**Only L5**

**PcbL5Ben_2181**	237 bp	**202 (100)**	3 (1.5)	3 (1.5)	3 (1.5)	Not in Mycobrowser	**Hypothetical protein, possibly IS256 transposase** (90% id, NCBI)	IS256 transposase is implicated in resistance to antibiotics and virulence (recombination for adaptation, invasion)(Murugesan et al. 2018; Gu et al. 2005)	L5, L6, bovis
**PcbL5Ben_2182**	897 bp	**202 (100)**	6 (3)	6 (3)	6 (3)	**Unknown function (Mb2048c**) Belongs to *RvD1* region	*Mb2048c* (belongs to *RvD1* region)	Function unknown (expressed during exponential growth in Sauton's minimal media (NCBI)). Gene absent in H37Rv (Mycobrowser)	L5, L6, bovis
**PcbL5Ben_3555**	1137 bp	164 (81.2)				Intermediary metabolism and respiration	moaA3 (NCBI)	*moaA3* (molybdenum cofactor biosynthesis protein subunit MoaA , Cyclic pyranopterin monophosphate synthase (Prokka)) is contained in an IS6110 sequence deleted in H37Rv (Mycobrowser)	L5, L6, bovis
**PcbL5Ben_3556**	1146 bp	162 (80.2)				Not in Mycobrowser	Hypothetical protein, possibly DnrI superfamily (NCBI)	*DnrI*: DNA binding transcriptional activator of the SARP family (signal transduction mechanisms, COG3629 (NCBI))	L5, L6, bovis
**PcbL5Ben_3557**	438 bp	162 (80.2)				Peptide synthase (Prokka)	Hypothetical protein, possibly IS6110 transposase (99-100% id NCBI)		L5, L6, bovis
**PcbL5Ben_3669**	396 bp	198 (98)				PE/PPE	Hypothetical protein		L5, L6

Ref: reference ; bp: base pair

*In total 207 in the 202 L5 Illumina sequenced genomes: more than one SNP in the gene for some strains

Present in L5 only, not present in L6 nor *M. bovis.* Those two genes (PcbL5Ben_2129 and 2130) are adjacent.

PcbL5Ben_1364 and PcbL5Ben_1365 are adjacent genes

PcbL5Ben_2181 and PcbL5Ben_2182 are adjacent genes

PcbL5Ben_3555, PcbL5Ben_3556 and PcbL5Ben_3557 are adjacent genes

Table S3. Presence/absence in 202 Illumina sequenced L5 genomes of the nine genes present in the H37Rv genome but absent in the three completed L5 genomes. The 4 genes absent in all L5 strains but present in H37Rv are presented in the grey-background lines of the table.

Genes present in H37Rv but absent in L5 completed PacBio sequenced genomes	Length (bp)	Present in L5 isolates Illumina sequenced genomes n=202 (%)	Absent in L5 isolates Illumina sequenced genomes n=202 (%)	Gene function			Absent in complete genome of	Present in complete genome of
				**Functional group (Mycobrowser)**	**Genes**	**Summary of role based on the literature**		
**Part of Rv1899c (H37Rv_2002)**	105 bp	201 (99.5)	1 (0.5)	Hypothetical protein (35 aa) part of *Rv1899c* (343 aa, Cell wall and cell processes)(Tuberculist)	Hypothetical protein, part of Rv1899c (LppD: 4-hydroxybuturate dehydrogenase 173 aa) and also LpqI beta-hexoaminidase precursor (Herrmann et al. 2000)	Possibly bacterial membrane lipoprotein (LppD and LpqI)	L5, L6, bovis	**H37Rv only**
**Rv1977 (H37Rv_2085)**	1047 bp	**0**	**202 (100)**	**Conserved hypotheticals**	M48 peptidase family protein including as homologs:<br>CAAX prenyl protease (*Htpx* (COG0501 O, heat shock stress response protein)<br>M48-Ste24p-like	Bacterial survival (bacterial self-degradation to eliminate abnormal membrane proteins, htpx upregulated at high temperature, and also after 96 hours of starvation (NCBI))	**L5 only**	L6, bovis, H37Rv
**Rv1978 (H37Rv_2086)**	849 bp	2 (1)	200 (99)	Conserved hypotheticals,	M48-2C-Ste24p (100% id), class I SAM-dependent methyltransferase (99.65% id), type 11 methyltransferase (97.1% id)	Bacterial survival in macrophages, and non-essential for in vitro growth of H37Rv (Rengarajan, Bloom, and Rubin 2005)(Mycobrowser, Tuberculist)	L5 only	L6, bovis, H37Rv
**Rv1979c (H37Rv_2087)**	1446 bp	**0**	**202 (100)**	**Cell wall and cell processes**	APC family permease	-Involved in the transport of **clofazimine** (and **bedaquiline**),	**L5 only**	L6, bovis, H37Rv

						associated with clofazimine and bedaquiline resistance (Zhang et al. 2015; Ghodousi et al. 2019)-Disruption of the gene provides an in vitro growth advantage to H37Rv		
**Rv1993c (H37Rv_2107)**	273 bp	**0**	**202 (100)**	**Conserved hypotheticals**	*Rv0968=DUF1490* similar to *Rv1993c* (NCBI)	Similar to *Rv0968* which is included in the operon *cosR-Rv0968-ctpV* where *cosR* and *ctpV* are virulence associated (Rademacher and Masepohl 2012)	**L5 only**	L6, bovis, H37Rv
**Rv1994c (H37Rv_2108)**	357 bp	1 (0.5)	201 (99.5)	Regulatory proteins	HTH transcriptional regulator *cmtR*	Operon *cmtR-Rv1993c-ctpG* is similar to *csoR-Rv0968-ctpV*.<br><br>*cmtR* (*Rv1994c*) has the same role as *csoR*.<br><br>These operons all function in the regulation and transport (efflux) of toxic metal especially copper which is toxic in excess (Samanovic et al. 2012).<br><br>Disruption may hamper in vitro growth, and survival during chronic phase of infection as *csoR* absence (Marcus et al. 2016; Rowland and Niederweis 2012; Ward, Hoye, and Talaat 2008).	L5 only	L6, bovis, H37Rv
**Rv1995 (H37Rv_2109)**	729 bp	**0**	**202 (100)**	**Conserved hypothetical**	**Hemerythrin-domain containing protein (NCBI)**	- Involved in oxygen transport (NCBI)	**L5 only**	L6, bovis, H37Rv
**Rv2073c (H37Rv_2188)**	750 bp	**0**	**202 (100)**	**Intermediary metabolism and respiration**	**Probable short chain dehydrogenase**	SDR family NAD(P)-dependent oxidoreductase (NCBI): catalyzes a wide range of reactions and substrate(Kavanagh et al. 2008; Sellés Vidal et al. 2018)	L5, L6, bovis	**H37Rv only**

**Rv2074 (H37Rv_2189)**	408 bp	0 (0)	**202 (100)**	**Intermediary metabolism and respiration (Pyridoxamine, Vit B6)**	**Pyridoxamine-5-phosphate oxidase (Mycobrowser, Tuberculist, NCBI)<br>Its cofactor (Selengut and Haft 2010) F420 dependent biliverdin reductase (99.2-100% id, NCBI)(Ahmed et al. 2016)**	- Biosynthesis of (pyridoxal phosphate and) pyridoxine (vitamin B6, Mycobrowser) which is essential for survival and virulence of M. tuberculosis (Dick et al. 2010)<br>- Its cofactor implicated in Immuno-evasive mechanism to allow bacterial persistence (Ahmed et al. 2016; Selengut and Haft 2010)	L5, L6, bovis	H37Rv only

Table S4. Verification of genes in the L5 completed PacBio sequenced genomes suspected to be pseudogenes in *M. tuberculosis* H37Rv genome.

Gene	Gene in PcbL5Ben	Gene in PcbL5Gam	Gene in PcbL5Nig	Length (aa)	Locus in H37Rv	Remarks	Conclusion
**1**	**PcbL5Ben_00749**	**PcbL5Gam_00750**	**PcbL5Nig_00748**	101	798857-799150	M1V, L96p, H97t, del NCD (aa 99-101)	**Pseudogene in H37Rv**
**2**	PcbL5Ben_00828	PcbL5Gam_00826	PcbL5Nig_00824	34	863155-863256	No change	Not pseudogene in H37Rv
**3**	Not suspected pseudogene	PcbL5Gam_00955	PcbL5Nig_00953	204	993212-992602	Inverted, M1V	**Pseudogene in H37Rv**
**4**	PcbL5Ben_01064	Not suspected pseudogene	Not suspected pseudogene	34	1102546-1102647	S23r	Not pseudogene in H37Rv
**5**	**PcbL5Ben_01108**	**PcbL5Gam_01103**	**PcbL5Nig_01100**	70	1148218-1148397	M1V	**Pseudogene in H37Rv**
**6**	**PcbL5Ben_01889**	**PcbL5Gam_01879**	**PcbL5Nig_01846**	68	1981341-1981341	M1V	**Pseudogene in H37Rv**
**7**	**PcbL5Ben_02128**	**PcbL5Gam_02111**	**PcbL5Nig_02084**	92	2219418-2219318	Inverted, alignment showed aa 59-92, no mutation	**Pseudogene in H37Rv**
**8**	Not suspected pseudogene	Not suspected pseudogene	PcbL5Nig_01853 is PcbNig_1896	41	2038813-2038706	Inverted, alignment showed aa 59-92, no mutation	**Pseudogene in H37Rv**
**9**	PcbL5Ben_02486	Not suspected pseudogene	Not suspected pseudogene	41	2582440-2582318	Inverted, M1V	**Pseudogene in H37Rv**
**10**	**PcbL5Ben_03141**	**PcbL5Gam_03117**	**PcbL5Nig_03092**	111	3291532-3291350	Inverted, del aa 1-aa60 (60 first aa)	**Pseudogene in H37Rv**
**11**	Not suspected pseudogene	Not suspected pseudogene	PcbL5Nig_02119	46	2256620-2256483	Inverted, No change	**Pseudogene in H37Rv**
**12**	Not suspected pseudogene	Not suspected pseudogene	PcbL5Nig_02291	33	24345567-2434665	No change	Not pseudogene in H37Rv
**13**	Not suspected pseudogene	Not suspected pseudogene	PcbL5Nig_02467	94	2614352-2614071	Inverted	**Pseudogene in H37Rv**
**14**	PcbL5Ben_03113	Not suspected pseudogene	PcbL5Nig_03060	204	3232652-3232867	Del aa1-aa56, N74D, del aa129-aa204	**Pseudogene in H37Rv**
**15**	Not suspected pseudogene	PcbL5Gam_03308	Not suspected pseudogene	58	3487871-3487698	Inverted, M1V	**Pseudogene in H37Rv**
**16**	**PcbL5Ben_03667**	**PcbL5Gam_03641**	**PcbL5Nig_03614**	99	3841840-3842109	M1V, all aa mutated (insertion substitution) from aa26-aa81, del aa82-aa98	**Pseudogene in H37Rv**

Table S5. Verification of genes present in H37Rv suspected to be pseudogenes in the L5 completed genomes

Gene in H37Rv	Length (aa)	Locus in H37Rv	Locus in PcbL5Ben	Locus in PcbL5Gam	Locus in PcbL5Nig	Remarks	Conclusion
H37Rv_02463	42	2604950-2605078	2631987-2632112	2614961-2615086	2603370-2603495	No change	Not pseudogene
**H37Rv_02556**	67	2718844-2719047	2743020-2742820	2729937-2729737	2716988-2716788	Inverted, nSNP I66L	**Pseudogene in the 3 Pacbio L5**
**H37Rv_02951**	55	3115741-3115908	3137926-3137762	3126133-3125969	3112866-3112702	Inverted, nSNP M1V, L55F	**Pseudogene in the 3 Pacbio L5**
**H37Rv_03797**	200	4053036-4053638	4076665-4077105	4064716-4065156	4055914-4056354	del aa 1-53	**Pseudogene in the 3 Pacbio L5**

Table S6. Genes absent in the Nigerian completed PacBio sequenced genome (PcbL5Nig) and some L5 isolates Illumina sequenced genomes (n=6), but present in the Benin and Gambian completed PacBio sequenced genomes (PcbL5Ben, PcbL5Gam), H37Rv and all other L5 isolates Illumina sequenced genomes.

ID (this study)	Rv number	Length (bp)	Functional group (Mycobrowser)	Gene name (Mycobrowser)	Product (Mycobrowser)	Function (Mycobrowser)
H37Rv_01580	*Rv1493*	2253 bp	Lipid metabolism	*mutB*	Probable methylmalonyl-CoA mutase large subunit MutB (MCM)	Involved in propionic acid fermentation
H37Rv_01581	*Rv1494*	303 bp	Virulence, detoxification, adaptation	*mazE4*	Possible antitoxin MazE4	Possible mazE4, antitoxin, part of toxin-antitoxin (TA) operon with Rv1495. Non-essential gene for in vitro growth of H37Rv
H37Rv_01582	*Rv1495*	318 bp	Virulence, detoxification, adaptation	*mazF4*	Possible toxin MazF4	Sequence-specific mRNA cleavage
H37Rv_01583	*Rv1496*	1005 bp	Cell wall and cell processes	*Rv1496*	Possible transport system kinase	Possibly involved in transport (possibly arginine)
H37Rv_01584	*Rv1497*	1290 bp	Intermediary metabolism and respiration	*lipL*	Probable esterase LipL	Function unknown, but supposed involvement in lipid metabolism
H37Rv_01585	*Rv1498c*	618 bp	Intermediary metabolism and respiration	*Rv1498c*	Probable methyltransferase	Causes methylation
H37Rv_01586	*Rv1498A*	213 bp	Conserved hypotheticals	*Rv1498A*	Conserved protein	Function unknown
	Rv1499	399 bp	Conserved hypotheticals	*Rv1499*	Hypothetical protein	Function unknown
H37Rv_01587	*Rv1500*	1029 bp	Intermediary metabolism and respiration	*Rv1500*	Probable glycosyltransferase	Function unknown
H37Rv_01588	*Rv1501*	822 bp	Conserved hypotheticals	*Rv1501*	Conserved hypothetical protein	Function unknown
H37Rv_01589	*Rv1502*	900 bp	Unknown	*Rv1502*	Hypothetical protein	Function unknown
H37Rv_01590	*Rv1503c*	549 bp	Conserved hypotheticals	*Rv1503c*	Conserved hypothetical protein	Function unknown

H37Rv_01591	*Rv1504*	600 bp	Conserved hypotheticals	*Rv1504c*	Conserved hypothetical protein	Function unknown
H37Rv_01592	*Rv1505c*	666 bp	Conserved hypotheticals	*Rv1505c*	Conserved hypothetical protein	Function unknown. It has some similarity to hypothetical proteins and glycosylases
H37Rv_01593	*Rv1506c*	501 bp	Unknown	*Rv1506c*	Hypothetical protein	Function unknown. Non-essential gene for in vitro growth of H37Rv
H37Rv_01594	*Rv1507A*	504 bp	Unknown	*Rv1507A*	Hypothetical protein	Function unknown
H37Rv_01595	*Rv1507c*	696 bp	Conserved hypotheticals	*Rv1507A*	Hypothetical protein	Function unknown
H37Rv_01596	*Rv1508c*	1800 bp	Cell wall and cell processes	*Rv1508c*	Probable membrane protein	Function unknown. Predicted to be in the GT-C superfamily of glycosyltransferases
H37Rv_01597	*Rv1508A*	636 bp	Conserved hypotheticals	*Rv1508A*	Conserved hypotheticals	Function unknown. Highly similar to central part of glycosyl transferases from various mycobacteria and eubacteria
H37Rv_01598	*Rv1509*	882 bp	Unknown	*Rv1509*	Hypothetical protein	Function unknown
H37Rv_01599	*Rv1510* (present in PcbL5Nig but missing in the PcbL5Nig-like Illumina L5)	1299 bp	Cell wall and cell processes	*Rv1510*	Probable conserved membrane protein	Function unknown
H37Rv_01600	*Rv1511*	1023 bp	Intermediary metabolism and respiration	*gmdA*	GDP-D-mannose dehydratase GmdA (GDP-mannose 4,6 dehydratase)	Function unknown, probably involved in nucleotide-sugar metabolism
H37Rv_01601	*Rv1512*	969 bp	Intermediary metabolism and respiration	*epiA*	Probable nucleotide-sugar epimerase EpiA	Function unknown, probably involved in nucleotide-sugar metabolism

H37Rv_01602	*Rv1513*	732 bp	Conserved hypotheticals	*Rv1513*	Conserved protein	Function unknown, similar to hypothetical proteins from several organisms
H37Rv_01603	*Rv1514c*	789 bp	Conserved hypotheticals	*Rv1514c*	Conserved hypothetical protein	Function unknown; similar to other hypothetical protein and to putative colanic acid biosynthesis glycosyl transferase
H37Rv_01604	*Rv1515c*	897 bp	Conserved hypotheticals	*Rv1515c*	Conserved hypothetical protein	Function unknown
H37Rv_01605	*Rv1516c*	1011 bp	Intermediary metabolism and respiration	*Rv1516c*	Probable sugar transferase	Function unknown, involved in cellular metabolism. Non essential for in vitro growth.
H37Rv_01606	*Rv1517*	765 bp	Cell wall and cell processes	*Rv1517*	Conserved hypothetical membrane protein	Function unknown
H37Rv_01607	*Rv1518*	960 bp	Conserved hypotheticals	*Rv1518*	Conserved hypothetical protein	possibly glycosyl transferase involved in exopolysaccharide synthesis, similar to several hypothetical proteins and glycosyl transferases from diverse organisms
H37Rv_01608	*Rv1519*	270 bp	Conserved hypotheticals	*Rv1519*	Conserved hypothetical protein	Function unknown
H37Rv_01609	*Rv1520*	1041 bp	Intermediary metabolism and respiration	*Rv1520*	Probable sugar transferase	Function unknown; thought to be involved in cellular metabolism
H37Rv_01610	*Rv1521*	1752 bp	Lipid metabolism	*fadD25*	Probable fatty-acid-AMP ligase FadD25 (fatty-acid-AMP synthetase) (fatty-acid-AMP synthase)	Function unknown; involved in lipid degradation
H37Rv_01611	*Rv1522c* (present in PcbL5Nig but missing in the PcbL5Nig-like Illumina L5)	3441 bp	Cell wall and cell processes	*mmpL12*	Probable conserved transmembrane transport protein MmpL12	Function unknown. Thought to be involved in fatty acid transport.

References for supplementary materials

Ahmed, F Hafna, A Elaaf Mohamed, Paul D Carr, Brendon M Lee, Karmen Condic-Jurkic, Megan L O'mara, and Colin J Jackson. 2016. "Rv2074 Is a Novel F 420 H 2-Dependent Biliverdin Reductase in Mycobacterium Tuberculosis." https://doi.org/10.1002/pro.2975.

Dick, Thomas, Ujjini Manjunatha, Barbara Kappes, and Martin Gengenbacher. 2010. "Vitamin B6 Biosynthesis Is Essential for Survival and Virulence of Mycobacterium Tuberculosis." *Molecular Microbiology* 78 (4): 980–88. https://doi.org/10.1111/j.1365-2958.2010.07381.x.

Gao, Juehua, Jie Liao, and Guang-Yu Yang. 2009. "Review Article CAAX-Box Protein, Prenylation Process and Carcinogenesis." *Am J Transl Res*. Vol. 1. www.ajtr.org/AJTR905003.

Ghodousi, Arash, Alamdar Hussain Rizvi, Aurangzaib Quadir Baloch, Abdul Ghafoor, Faisal Masood, Mehmood Qadir, Emanuele Borroni, Alberto Trovato, Sabira Tahseen, and Daniela Maria. 2019. "Acquisition of Cross-Resistance to Bedaquiline and Clofazimine Following Treatment for Tuberculosis in 2 Pakistan." https://doi.org/10.1101/630012.

Gu, J, H Li, M Li, C Vuong, M Otto, Y Wen, and Q Gao. 2005. "Bacterial Insertion Sequence IS256 as a Potential Molecular Marker to Discriminate Invasive Strains from Commensal Strains of Staphylococcus Epidermidis." *The Journal of Hospital Infection* 61 (4): 342–48. https://doi.org/10.1016/j.jhin.2005.04.017.

Herrmann, Jean Louis, Robin Delahay, Alex Gallagher, Brian Robertson, and Douglas Young. 2000. "Analysis of Post-Translational Modification of Mycobacterial Proteins Using a Cassette Expression System." *FEBS Letters* 473 (3): 358–62. https://doi.org/10.1016/S0014-5793(00)01553-2.

Kavanagh, K L, H Jçrnvall, B Persson, and U Oppermann. 2008. "The SDR Superfamily: Functional and Structural Diversity within a Family of Metabolic and Regulatory Enzymes" 171: 77. https://doi.org/10.1007/s00018-008-8588-y.

Marcus, Sarah A, Sarah W Sidiropoulos, Howard Steinberg, and Adel M Talaat. 2016. "CsoR Is Essential for Maintaining Copper Homeostasis in Mycobacterium Tuberculosis." https://doi.org/10.1371/journal.pone.0151816.

Murugesan, Saravanan, Stalin Mani, Indhumathy Kuppusamy, and Padma Krishnan. 2018. "Role of Insertion Sequence Element Is256 as a Virulence Marker and Its Association with Biofilm Formation among Methicillin-Resistant Staphylococcus Epidermidis from Hospital and Community Settings in Chennai, South India." *Indian Journal of Medical Microbiology* 36 (1): 124. https://doi.org/10.4103/ijmm.IJMM_17_276.

Rademacher, Corinna, and Bernd Masepohl. 2012. "Copper-Responsive Gene Regulation in Bacteria." https://doi.org/10.1099/mic.0.058487-0.

Rengarajan, Jyothi, Barry R Bloom, and Eric J Rubin. 2005. "Genome-Wide Requirements for Mycobacterium Tuberculosis Adaptation and Survival in Macrophages." *PNAS June*. Vol. 7. www.pnas.orgcgidoi10.1073pnas.0503272102.

Rowland, Jennifer L, and Michael Niederweis. 2012. "Resistance Mechanisms of Mycobacterium Tuberculosis against Phagosomal Copper Overload." https://doi.org/10.1016/j.tube.2011.12.006.

Samanovic, Marie I., Chen Ding, Dennis J. Thiele, and K. Heran Darwin. 2012. "Copper in Microbial Pathogenesis: Meddling with the Metal." *Cell Host & Microbe* 11 (2): 106–15. https://doi.org/10.1016/j.chom.2012.01.009.

Selengut, Jeremy D, and Daniel H Haft. 2010. “Unexpected Abundance of Coenzyme F 420-Dependent Enzymes in Mycobacterium Tuberculosis and Other Actinobacteria †.” *JOURNAL OF BACTERIOLOGY* 192 (21): 5788–98. https://doi.org/10.1128/JB.00425-10.

Sellés Vidal, Lara, Ciarán L. Kelly, Paweł M. Mordaka, and John T. Heap. 2018. “Review of NAD(P)H-Dependent Oxidoreductases: Properties, Engineering and Application.” *Biochimica et Biophysica Acta (BBA) - Proteins and Proteomics* 1866 (2): 327–47. https://doi.org/10.1016/j.bbapap.2017.11.005.

Ward, Sarah K, Elizabeth A Hoye, and Adel M Talaat. 2008. “The Global Responses of Mycobacterium Tuberculosis to Physiological Levels of Copper †.” *JOURNAL OF BACTERIOLOGY* 190 (8): 2939–46. https://doi.org/10.1128/JB.01847-07.

Zhang, Shuo, Jiazhen Chen, Peng Cui, Wanliang Shi, Wenhong Zhang, and Ying Zhang. 2015. “Identification of Novel Mutations Associated with Clofazimine Resistance in Mycobacterium Tuberculosis.” https://doi.org/10.1093/jac/dkv150.

Table S7. Sublineage information for all included isolates

L5 strains	Sublineage	Remark
PcbL5Benin	L5.3 (L5.3.1)	
PcbL5Gambia	L5.1.5	
PcbL5Nigeria	L5.3 (L5.3.2)	
10451-01	L5.1.1	
10463-02	L5.1.1	
10480-01	L5.1.1	
10519-01	L5.1.1	
10617-12	L5.1.1	
10695-13	unknown (new sublineage)	
10709-13	L5.1.1	
11821-03	unknown (new sublineage)	lacks RD711
BSSE-QGF-22150	L5.1.3	
BSSE-QGF-22151	L5.1.1	
BSSE-QGF-22153	L5.1.1	
BSSE-QGF-22154	L5.1.4	
BSSE-QGF-22155	L5.1.1	
BSSE-QGF-27970	L5.1.1	
BSSE-QGF-28835	L5.3 (L5.3.2)	
BSSE-QGF-54153	unknown (new sublineage)	
BSSE-QGF-78571	L5.1.4	
DRC-072384	L5.2	
ERR019875	L5.1.2	
ERR1023216	unknown (new sublineage)	lacks RD711
ERR1023217	L5.1.3	
ERR1023218	unknown (new sublineage)	lacks RD711
ERR1023221	L5.3 (L5.3.2)	
ERR1023223	L5.1.5	
ERR1023224	L5.1.2	
ERR1082117	L5.1.1	
ERR1082122	L5.1.2	
ERR1082125	L5.1.1	
ERR1082126	L5.1.1	
ERR1082129	L5.1.2	
ERR1082135	L5.1.1	
ERR1082137	L5.1.3	
ERR1203054	L5.1.1	
ERR1203057	L5.1.1	
ERR1203058	L5.1.2	
ERR1203059	L5.1.1	
ERR1203065	L5.1.1	
ERR1203066	L5.1.2	
12026-12	L5.1.1	
1447-02	unknown (new sublineage)	lacks RD711
4802-03	L5.1.4	
5438-02	L5.1.1	
7491-14	L5.1.1	
BSSE-QGF-22149	L5.1.2	
BSSE-QGF-54138	L5.1.4	
ERR1082120	L5.1.1	
ERR1203068	L5.1.2	

ERR2383621	L5.1.1	
ERR439939	L5.1.4	
ERR439964	L5.1.1	
ERR502505	L5.1.1+ (Mixed infection)	Excluded from the analysis
ERR702407	L5.1.3	
ERR751308	L5.1.1	
ERR751348	L5.1.4	
ERR1203069	L5.1.4	
ERR1203074	L5.1.1	
ERR1215463	unknown (new sublineage)	
ERR1215473	L5.1.5	
ERR1215476	L5.1.4	
ERR1215477	L5.1.1	
ERR1215478	L5.1.2+ (Mixed infection)	Excluded from the analysis
ERR1334053	L5.1.1	
ERR234679	L5.1.3	
ERR234680	unknown (new sublineage)	
ERR2383619	L5.1.1	
ERR2383620	L5.2	
ERR2383622	L5.3 (L5.3.1)	
ERR2383623	L5.2	
ERR2383624	L5.2	
ERR2383625	L5.1.1	
ERR2383626	L5.2	
ERR2704808	L5.1.1	
ERR2704809	L5.1.1	
ERR2704810	L5.2	
ERR2704812	L5.2	
ERR439931	L5.1.3	
ERR439936	L5.1.5	
ERR439937	L5.1.2	
ERR439940	L5.1.5	
ERR439941	L5.1.4	
ERR439944	unknown (new sublineage)	
ERR439947	L5.1.5	
ERR439949	L5.1.4	
ERR439951	L5.3 (L5.3.2)	
ERR439952	L5.3 (L5.3.1)	
ERR439953	L5.1.5	
ERR439955	L5.1.4	
ERR439959	L5.3 (L5.3.2)	
ERR439960	unknown (new sublineage)	
ERR439962	unknown (new sublineage)	
ERR439967	L5.1.1	
ERR439980	L5.1.1	
ERR439982	unknown (new sublineage)	
ERR439983	L5.3 (L5.3.1)	
ERR439984	unknown (new sublineage)	
ERR439985	unknown (new sublineage)	
ERR460916	L5.3 (L5.3.1)	
ERR502471	L5.1.1	
ERR502475	L5.1.1	

ERR502487	unknown (new sublineage)	lacks RD711
ERR502500	L5.1.1	
ERR502501	L5.3 (L5.3.2)	
ERR502509	L5.1.5	
ERR502512	L5.3 (L5.3.2)	
ERR502513	L5.1.4	
ERR502515	L5.1.1	
ERR502536	L5.1.4	
ERR550904	L5.2	
ERR551005	L5.2	
ERR551566	unknown (new sublineage)	lacks RD711
ERR551965	L5.1.1	
ERR552187	L5.1.1	
ERR552345	L5.1.1	
ERR702402	L5.1.1	
ERR702411	L5.1.1	
ERR702413	unknown (new sublineage)	lacks RD711
ERR702416	unknown (new sublineage)	
ERR702417	L5.1.1	
ERR702419	L5.3 (L5.3.1)	
ERR702426	L5.1.1	
ERR751295	L5.1.4	
ERR751299	L5.1.1	
ERR751301	L5.1.1	
ERR751302	L5.1.1+ (Mixed infection)	Excluded from the analysis
ERR751303	L5.1.1	
ERR751304	L5.1.3	
ERR751310	L5.1.3	
ERR751311	L5.1.5	
ERR751312	L5.3 (L5.3.1)	
ERR751313	L5.1.1	
ERR751315	L5.1.3	
ERR751323	L5.1.4	
ERR751327	L5.1.1	
ERR751328	L5.1.1	
ERR751334	L5.1.1	
ERR751335	unknown (new sublineage)	lacks RD711
ERR751339	L5.1.2	
ERR751345	L5.1.1	
12046-12	L5.1.5	
12184-03	unknown (new sublineage)	lacks RD711
12910-13	L5.1.1	
1410-02	L5.1.3	
1413-02	L5.1.1	
1426-02	L5.1.1	
1437-02	L5.1.1	
1440-02	L5.1.1	
1465-02	L5.1.4	
1473-02	L5.1.4	
14741-14	L5.1.3	
2568-02	unknown (new sublineage)	
3377-03	L5.1.1	

3482-03	L5.1.4	
4005-13	L5.1.1	
4518-03	L5.1.1	
4804-03	L5.1.1	
5378-02	L5.1.1	
5398-02	L5.1.1	
5404-02	L5.1.1	
5405-02	unknown (new sublineage)	lacks RD711
5417-02	L5.1.2	
5432-02	L5.1.2	
5434-02	L5.1.4	
5441-04	L5.1.1	
5444-02	L5.2	
5446-02	unknown (new sublineage)	lacks RD711
5447-02	L5.1.1	
5456-02	L5.1.1	
5473-02	L5.1.4	
5475-02	L5.1.1	
6897-04	unknown (new sublineage)	lacks RD711
8076-11	L5.2	
8103-11	L5.2	
8137-11	L5.2	
8214-03	L5.1.1	
8270-03	L5.1.1	
8303-02	L5.1.1	
8876-12	L5.1.1	
8878-12	L5.1.1	
8946-13	L5.1.1	
9858-03	L5.3 (L5.3.1)	
BSSE-QGF-108892	L5.1.1	
BSSE-QGF-109560	L5.1.1	
BSSE-QGF-109571	L5.1.1	
BSSE-QGF-109627	L5.1.1	
BSSE-QGF-22108	unknown (new sublineage)	lacks RD711
BSSE-QGF-22110	L5.1.3	
GR2-CGATGT-L007	L5.1.1	
MTB-DY-135	L5.1.2	
MTB-DY-20	unknown (new sublineage)	lacks RD711
MTB-DY-26	L5.3 (L5.3.1)	
NG-5288-1047301	L5.1.1	
NG-5288-256902	L5.1.1	
NG-5288-533304	L5.1.1	
NG-5288-553604	L5.1.1	
NG-5967-N1063	unknown (new sublineage)	lacks RD711
NG-6193-N1203	L5.1.1	
SRR2100183	L5.1.2	
SRR2100713	unknown (new sublineage)	lacks RD711
SRR2101040	L5.1.2	
SRR2101063	L5.1.2	
SRR2101065	L5.1.4	
SRR2101293	L5.1.1	
SRR7496542	unknown (new sublineage)	lacks RD711

SRR998585	L5.1.1	
SRR998618	L5.1.1	

Table S8. Accession numbers, country of origin and sequencing statistics of Illumina sequenced L5 genomes

G_NUMBER	AC_NUMBER	Country	Avg. read depth	Std. read depth	% uncovered
G00183	ERR234142	Ghana	80.93	39.07	2.27%
G00188	ERR234144	Ghana	76.54	29.9	1.65%
G00193	ERR234147	Ghana	83.46	27.11	0.47%
G00196	ERR234149	Ghana	87.75	28.61	0.63%
G00410	ERR841430	Liberia	82.95	17.69	0.81%
G00731	ERR4192047	Ghana	62.29	14.16	0.72%
G00829	ERR841494	South Africa	107.03	26.57	0.70%
G00892	ERR234199	Ghana	131.33	38.69	0.89%
G00895	ERR234202	Ghana	32.72	11.7	1.49%
G00898	ERR234204	Ghana	60.94	17.86	1.39%
G01935	ERR4192364	Nigeria	73.63	21.26	0.69%
G01937	ERR4192366	Nigeria	56.86	17.92	0.91%
G01976	ERR4192367	Equatorial Guinea	105.69	46.37	0.55%
G01977	ERR4192368	Equatorial Guinea	89.16	21.3	0.68%
G01978	ERR4192369	Equatorial Guinea	91.39	25.77	0.57%
G01979	ERR4192370	Equatorial Guinea	145.14	36.5	0.73%
G01980	ERR4192371	Equatorial Guinea	61.55	30.86	0.57%
G01981	ERR4192372	Equatorial Guinea	85.46	30.78	0.69%
G01982	ERR4192373	Equatorial Guinea	68.64	34.69	0.73%
G02691	ERR4192380	Gambia	125.46	32.27	1.23%
G03829	ERR1334053	Ghana	96.04	17.71	0.94%
G04163	ERR1215478	Ghana	32.19	8.67	0.61%
G04164	ERR1215477	Ghana	50.23	10.28	0.58%
G04165	ERR1215476	Ghana	55.72	11.26	0.60%
G04168	ERR552345	Outside Africa	65.65	29.25	0.64%
G07450	ERR4192561;ERR4192562; ERR4192563	Ivory Coast	86.37	26.22	0.58%
G08166	ERR4192386;ERR4192407; ERR4192544	Equatorial Guinea	61.68	20.98	0.73%
G08388	ERR1023216	Gambia	147.25	26.27	0.72%
G08389	ERR1023217	Gambia	141.73	27	0.89%
G08390	ERR1023218	Gambia	150.36	27.06	0.69%
G08393	ERR1023221	Gambia	154.28	31.33	1.09%
G08395	ERR1023223	Gambia	152.6	27.95	0.48%
G08396	ERR1023224	Gambia	146.32	31.4	0.64%

G08470	ERR1082117	Ghana	177.17	34.08	0.71%
G08472	ERR1082120	Ghana	178.32	42.5	0.57%
G08473	ERR1082122	Ghana	162.75	32.02	0.54%
G08476	ERR1082125	Ghana	176.1	46.33	0.59%
G08477	ERR1082126	Ghana	141.32	25.18	0.61%
G08480	ERR1082129	Ghana	188.23	28.52	0.56%
G08486	ERR1082135	Ghana	151.2	25.51	0.93%
G08488	ERR1082137	Ghana	160.3	26.42	0.81%
G08496	ERR1203054	Ghana	127.36	21.51	0.77%
G08500	ERR1203057	Ghana	119.35	22.27	0.56%
G08501	ERR1203058	Ghana	119.66	23.45	0.57%
G08502	ERR1203059	Ghana	112.83	19.47	0.59%
G08507	ERR1203065	Ghana	112.84	18.2	0.56%
G08508	ERR1203066	Ghana	124.55	19.6	0.57%
G08510	ERR1203068	Ghana	123.36	20.14	0.52%
G08511	ERR1203069	Ghana	112.67	20.24	0.94%
G08516	ERR1203074	Ghana	125.48	20.35	0.72%
G08541	ERR439931	Cameroon	79.93	13.46	0.05%
G08546	ERR439936	Guinea	83.16	14.05	0.51%
G08547	ERR439937	Benin	64.58	11.92	0.55%
G08549	ERR439939	Benin	57.11	11.07	0.60%
G08550	ERR439940	Benin	65.84	12.15	0.67%
G08551	ERR439941	Benin	67.66	11.61	0.41%
G08554	ERR439944	Benin	74.03	12.89	0.46%
G08557	ERR439947	Benin	64.2	11.79	0.50%
G08559	ERR439949	Benin	65.95	12.04	0.59%
G08561	ERR439951	Benin	78.95	14.69	1.16%
G08562	ERR439952	Benin	75.54	13.56	0.49%
G08563	ERR439953	Benin	70.79	12.12	0.60%
G08565	ERR439955	Benin	66.89	12.8	1.16%
G08569	ERR439959	Benin	60.49	13.01	1.11%
G08570	ERR439960	Benin	58.87	10.94	0.44%
G08572	ERR439962	Benin	62.57	11.35	0.63%
G08574	ERR439964	Benin	64.07	12.74	0.70%
G08577	ERR439967	Guinea	68.33	12.44	0.55%
G08590	ERR439980	Ivory Coast	64.44	11.89	0.54%
G08592	ERR439982	Benin	53.23	10.36	0.74%
G08593	ERR439983	Benin	65.41	13.82	0.71%
G08594	ERR439984	Benin	70.69	13.36	0.57%
G08595	ERR439985	Benin	63.36	11.61	0.40%
G08609	ERR460916	Benin	93.26	16.62	0.45%
G08611	ERR502471	Ghana	79.03	13.94	0.63%

G08612	ERR502475	Ghana	95.84	15.31	0.17%
G08616	ERR502487	Ghana	82.11	13.94	0.72%
G08619	ERR702402	Ghana	76.42	14.85	0.73%
G08622	ERR502500	Ghana	80.33	13.65	0.69%
G08623	ERR502501	Ghana	99.81	18.96	1.08%
G08627	ERR502505	Ghana	89.13	17.2	0.11%
G08631	ERR502509	Ghana	96.96	16.51	0.52%
G08632	ERR502512	Ghana	89.75	16.35	0.61%
G08633	ERR502513	Ghana	54.44	14.2	0.92%
G08635	ERR502515	Ghana	80.8	15.83	0.66%
G08652	ERR502536	Ghana	77.92	14.2	0.91%
G08656	ERR1215473	Ghana	47.9	11.37	0.55%
G08660	ERR702407	Ghana	74.28	14.65	0.54%
G08664	ERR702411	Ghana	76.88	15.2	0.69%
G08666	ERR702413	Ghana	75.3	12.3	0.52%
G08669	ERR702416	Ghana	75.09	16.34	0.56%
G08670	ERR702417	Ghana	80.41	15.1	0.54%
G08672	ERR702419	Ghana	77.66	12.49	0.51%
G08679	ERR702426	Ghana	74.15	12.52	0.54%
G08695	ERR751295	Ghana	86.88	17.5	0.92%
G08699	ERR751299	Ghana	84.94	16.75	0.56%
G08701	ERR751301	Ghana	81.22	14.65	0.54%
G08702	ERR751302	Ghana	87.89	16.87	0.70%
G08703	ERR751303	Ghana	83.14	15.78	0.54%
G08704	ERR751304	Ghana	92.78	18.52	0.74%
G08708	ERR751308	Ghana	77.59	13.77	0.39%
G08710	ERR751310	Ghana	55.2	16.93	0.65%
G08711	ERR751311	Ghana	80.87	14.44	0.50%
G08712	ERR751312	Ghana	83.18	16.82	0.38%
G08713	ERR751313	Ghana	77.85	15.8	0.60%
G08715	ERR751315	Ghana	82.84	13.98	0.55%
G08721	ERR751323	Ghana	89.55	14.95	0.68%
G08723	ERR751327	Ghana	67.08	11.91	0.56%
G08724	ERR751328	Ghana	65.72	12.13	0.56%
G08730	ERR751334	Ghana	86.4	16.54	0.60%
G08731	ERR751335	Ghana	75.31	13.26	0.64%
G08735	ERR751339	Ghana	49.43	9.68	0.58%
G08741	ERR751345	Ghana	69.72	12.98	0.54%
G08743	ERR751348	Ghana	92.05	16.94	0.91%
G08848	ERR1215463	Ghana	44.99	9.31	0.52%
G08913	ERR019875	Outside Africa	29.23	11.6	1.64%
G08917	ERR234679	Outside Africa	126.4	20.86	0.85%

G08918	ERR234680	Outside Africa	116.97	19.1	0.51%
G08920	ERR551566	Sierra Leone	64.74	17.79	0.77%
G08934	SRR998585	Mali	151.95	32.17	0.12%
G11003	SRR998618	Mali	151.66	36.31	0.32%
G11556	ERR3170399	Ghana	52.65	14.83	0.86%
G11565	ERR3170440	Ghana	86.9	21.54	0.77%
G11566	ERR3170441	Ghana	44.99	13.94	1.19%
G11568	ERR4162017	Gabon	59.41	16.96	0.74%
G11575	ERR551089	Sierra Leone	66.53	19.82	1.12%
G11578	ERR3170443	Ghana	67.25	17.96	0.71%
G11579	ERR551857	Sierra Leone	61.23	17.31	0.76%
G11591	ERR3170456	Ghana	105.34	25.94	0.66%
G11595	ERR3170460	Ghana	45.89	12.79	1.01%
G11597	ERR3170463	Ghana	42.7	12.71	0.70%
G11598	ERR3170464	Ghana	122.54	34.01	0.85%
G11600	ERR3170466	Ghana	113.82	36.95	0.77%
G11604	ERR3170471	Ghana	47.59	13.62	1.02%
G11605	ERR552588	Ghana	73.32	20.22	1.06%
G11607	ERR3170473	Ghana	49.4	13.09	1.02%
G11608	ERR3170474	Ghana	91.38	23.93	0.69%
G11609	ERR3170475	Ghana	51.37	16.02	0.76%
G11610	ERR3170476	Ghana	65.15	18.85	0.71%
G11611	ERR3170477	Ghana	43.03	11.35	0.72%
G11614	ERR3170480	Ghana	67.64	23.17	0.83%
G11619	ERR3170483	Ghana	71.16	19.26	0.58%
G11620	ERR3170484	Ghana	105.94	26.91	0.75%
G11626	ERR3170490	Ghana	104.41	24.65	0.67%
G11627	ERR3170491	Ghana	49.54	13.68	0.73%
G11631	ERR4162025	Gabon	56.05	15.47	0.90%
G11632	ERR4162026	Gabon	102.51	29.46	0.85%
G11633	ERR4162027	Gabon	117.26	31.58	0.68%
G11637	ERR3148046	Ghana	81.18	21	0.50%
G11639	ERR4162032	Democratic Republic of the Congo	81.46	20.79	0.63%
G11643	ERR3170404	Ghana	104.28	28.22	0.96%
G11646	ERR3170407	Ghana	49.53	15.96	0.82%
G11649	ERR552187	Ghana	68.92	19.83	0.96%
G11654	ERR3170413	Ghana	75.04	20.49	0.87%
G11655	ERR4162000	Gabon	75.66	20.02	0.88%
G11656	ERR4162001	Gabon	83.32	23.6	0.56%
G11657	ERR4162002	Gabon	133.3	35.31	0.72%

G11658	ERR551336	Sierra Leone	64.78	17.85	0.78%
G11659	ERS4575215	Gabon	95.25	23.79	0.83%
G11660	ERR4162005	Gabon	79.69	21.03	0.88%
G11661	ERR3170414	Ghana	56.8	14.98	0.97%
G11663	ERR4162006	Gabon	97.1	27.08	0.69%
G11666	ERR3170417	Ghana	66.83	16.98	0.78%
G11667	ERR3170418	Ghana	64.88	16.11	0.80%
G11668	ERR3170419	Ghana	89.74	23.14	0.71%
G11671	ERR3170422	Ghana	117.23	28.65	0.65%
G11672	ERR3170423	Ghana	104.37	28.35	0.72%
G11674	ERR3170424	Ghana	117.45	32.15	0.72%
G11679	ERR3170428	Ghana	47.74	13.31	1.15%
G11683	ERR552506	Ghana	57.64	18.39	1.16%
G11685	ERR4162007	Gabon	119.87	32.72	0.80%
G11693	ERR3170434	Ghana	175.53	43.2	0.58%
G11696	ERR552261	Sierra Leone	63.95	18.67	0.85%
G11698	ERR3148058	Gabon	167.21	46.01	0.67%
G11700	ERR551005	Republic of the Congo	69.42	17.32	0.74%
G11701	ERR551124	Republic of the Congo	69.8	17.67	0.85%
G11702	ERR552673	Republic of the Congo	64.88	18.67	1.15%
G11703	ERR550904	Republic of the Congo	122.71	31.58	0.67%
G11704	ERR552285	Republic of the Congo	64.04	16.89	0.68%
G11706	ERR3170488	Ghana	90.82	29.02	0.61%
G14330	SRR2100183	Outside Africa	65.57	12.1	0.54%
G14855	SRR2100713	Outside Africa	51.05	12.71	0.33%
G15178	SRR2101040	Outside Africa	68.07	13.78	0.47%
G15201	SRR2101063	Outside Africa	55.9	13.93	0.75%
G15203	SRR2101065	Outside Africa	51.53	11.67	0.68%
G15430	SRR2101293	Outside Africa	55.26	12.58	0.62%
G22215	ERR2704692	Ghana	55.29	14.56	0.63%
G25357	ERR550904	Republic of the Congo	122.71	31.58	0.67%
G26731	SRR11444083	Ghana	55.19	11.16	0.63%
G26738	SRR11444026	Ghana	47.69	9.74	0.67%
G26749	SRR11444232	Ghana	62.94	10.52	0.42%
G26805	SRR11444206	Ghana	69.52	12.03	0.55%
G32940	ERR2383619	Cameroon	115.01	28.88	0.81%

G32941	ERR2383620	Cameroon	149.11	35.82	0.79%
G32942	ERR2383621	Ivory Coast	89.61	20.8	0.57%
G32943	ERR2383622	Outside Africa	82.08	21.12	0.51%
G32944	ERR2383623	Outside Africa	69.76	18.79	0.88%
G32945	ERR2383624	Outside Africa	83.77	22.73	0.86%
G32946	ERR2383625	Outside Africa	92.66	24.9	0.72%
G32947	ERR2383626	Outside Africa	82.86	20.7	0.54%
G32950	ERR2704808	Ivory Coast	327.61	42.04	0.60%
G32951	ERR2704809	Cameroon	316.26	40.72	0.58%
G32952	ERR2704810	Cameroon	329.55	40.39	0.41%
G32954	ERR2704812	Cameroon	378.7	59.3	1.35%
G33338	SRR7496542	Outside Africa	146.46	25.1	0.46%
